# Systemically injected bone marrow mononuclear cells specifically home to axially vascularized tissue engineering constructs

**DOI:** 10.1371/journal.pone.0272697

**Published:** 2022-08-11

**Authors:** Ahmad Eweida, Sophia Flechtenmacher, Elli Sandberg, Matthias Schulte, Volker J. Schmidt, Ulrich Kneser, Leila Harhaus

**Affiliations:** 1 Department of Hand, Plastic and Reconstructive Surgery, Burn Center, BG Trauma Center Ludwigshafen, University of Heidelberg, Ludwigshafen, Germany; 2 Department of Head, Neck and Endocrine Surgery, Faculty of Medicine, University of Alexandria, Alexandria, Egypt; 3 Department for Plastic and Breast Surgery, Zealand University Hospital, Roskilde, Copenhagen University, Copenhagen, Denmark; Goa University, India, INDIA

## Abstract

Inducing axial vascularisation of tissue engineering constructs is a well-established method to support tissue growth in large 3-dimensional tissues. Progenitor cell chemotaxis towards axially vascularized tissues has not been well characterized. In a prospective randomized controlled study including 32 male syngeneic Lewis rats we investigated the capability of the axially vascularized constructs to attract systemically injected bone marrow mononuclear cells (BMMNCs). The underlying mechanism for cell homing was investigated focusing on the role of hypoxia and the SDF1-CXCR4-7 axis. Sixteen animals were used as donors for BMMNCs. The other animals were subjected to implantation of a tissue engineering construct in the subcutaneous groin region. These constructs were axially vascularized either via an arteriovenous loop (AVL, n = 6) or via uninterrupted flow-through vessels (non-AVL, n = 10). BMMNCs were labelled with quantum dots (Qdot® 655) and injected 12 days after surgery either via intra-arterial or intravenous routes. 2 days after cell injection, the animals were sacrificed and examined using fluorescence microscopy. The Qdot® 655 signals were detected exclusively in the liver, spleen, AVL constructs and to a minimal extent in the non-AVL constructs. A significant difference could be detected between the number of labelled cells in the AVL and non-AVL constructs with more cells detected in the AVL constructs specially in central zones (*p* <0.0001). The immunohistological analysis showed a significant increase in the absolute expression of HIF-1 in the AVL group in comparison to the non-AVL group. The PCR analysis confirmed a 1.4-fold increase in HIF-1 expression in AVL constructs. Although PCR analysis showed an enhanced expression of CXCR4 and CXCR7 in AVL constructs, no significant differences in SDF1 expression were detected via immunohistological or PCR analysis. At the examined time point, the AVL constructs can attract BMMNCs in a mechanism probably related to the hypoxia associated with a robust tissue formation.

## Introduction

Tissue engineering and regenerative medicine are rapidly progressing as a promising reconstructive modality with the major advantage of avoiding donor site morbidity. Despite the progress in the techniques of regenerative medicine in the last decade, a major concern is still the ability to provide an adequate vascularisation for large 3-dimensional tissues [1]. Among the methods of providing vascularisation to tissue engineering constructs is the induction of axial vascularisation via arterio-venous loops (AVL) using standard microsurgical techniques [2–4]. This method of axial vascularisation has shown satisfying results in providing adequate blood supply to reconstruct critical size defects of several tissues both in animal and clinical studies and is currently a subject for further clinical trials [5–7].

The robust ability of the AVL to induce neo-vascularisation and tissue regeneration has been the focus of intensive research. Beside the already proven role of an induced high flow within the loop [8] and that of the controlled ischemia within the AVL-constructs in the initial phases [9], the role of the AVL as a chemotactic point for progenitor cells could be relevant but remains inadequately investigated. Our previous work on AVL- bone constructs proved their ability to induce bone formation even after applying a high dose of irradiation. This suggested a definite role of progenitor cell chemotaxis towards the constructs [10]. However, the native ability of the AVL-constructs to attract progenitor cells from the systemic circulation is still not well characterized. The aim of the current study is to explore the ability of AVL-constructs to specifically attract systemically injected bone marrow mononuclear cells (BMMNCs).

BMMNCs are a clinically convenient source for progenitor cells and are currently well established as a rich pool of progenitor cells for many clinical applications [11]. BMMNCs are a heterogeneous group of cells derived from the bone marrow containing B-cells, T-cells, monocytes, as well as rare progenitor cells such as hematopoietic stem cells, mesenchymal stromal cells, endothelial progenitor cells and very small embryonic-like cells representing a rich plethora of cells which interactively augment tissue regeneration and vascularisation [12]. Using the whole pool of BMMNCs in cell therapies does not require sophisticated techniques of *ex vivo* cell isolation and expansion and has shown advantages in several clinical trials including tissue repair after injury [11, 13]. BMMNC engraftment to target tissues after systemic injection remains however a challenge facing cell therapies [14]. One of the well-studied chemotactic axes regulating progenitor cell trafficking under physiological and pathological conditions is the hypoxia induced factor 1- (HIF-1)—stromal derived factor (SDF-1, also known as CXCL12) axis with its target receptors CXCR4 and CXCR7 (also known as atypical chemokine receptor, ACKR3) [15, 16]. In this work, we investigated the native capability of the AVL-constructs to specifically attract systemically injected BMMNCs. Studying the cell distribution of systemically injected BMMNCs would provide information about the cell behavior of endogenous BMMNCs in relation to AVL-constructs. In a similar setting to progenitor cell therapy, we studied the engraftment of the injected BMMNCs to the AVL-constructs in comparison to non-AVL constructs after both intra-arterial and intravenous cell injections. The underlying mechanism for this progenitor cell homing was also investigated focusing on the role of hypoxia and the SDF1-CXCR4-7 axis.

## Materials and methods

### Study design

A prospective randomized controlled study including 32 male syngeneic Lewis rats weighing 290–340 g (Charles River, Sulzfeld, Germany). Sixteen animals were used as donors for BMMNCs. The other 16 animals were subjected to implantation of a tissue engineering construct in the subcutaneous groin region. These constructs were axially vascularized either via an arteriovenous loop (AVL) or via uninterrupted flow-through vessels (non-AVL). Twelve days after implantation, all animals received a single injection of nanoparticle labelled BMMNCs either via intra-arterial or intravenous route as shown in Table 1. Two days after injection, the animals were sacrificed and the tissue distribution of the labelled BMMNC was analyzed in the implanted constructs and in various organs. Additionally, the expression of HIF-1alpha, SDF-1, CXCR4 and CXCR7 in the constructs was analyzed via immunofluorescence and RT-PCR analysis. The study was approved by the institutional animal care committee and the local governmental authorities (Landesuntersuchungsamt Rheinland-Pfalz, study registration number 23177–07 / G16-7-037).

**Table 1:** Study design.

Chamber implantation	BMMNC injection day 12 postoperative	Sacrifice and analysis day 14 postoperative
AVL construct (n = 6)	intravenous (n = 3) intra-arterial (n = 3)	fluorescence microscopy (lung, liver, spleen, kidney, bone marrow) Immunofluorescence (constructs) PCR analysis (constructs)
Non-AVL construct (n = 10)	intravenous (n = 5) intra-arterial (n = 5)	
	donor animals (n = 16)	

### Surgical implantation of tissue engineering constructs

**Fig 1:**
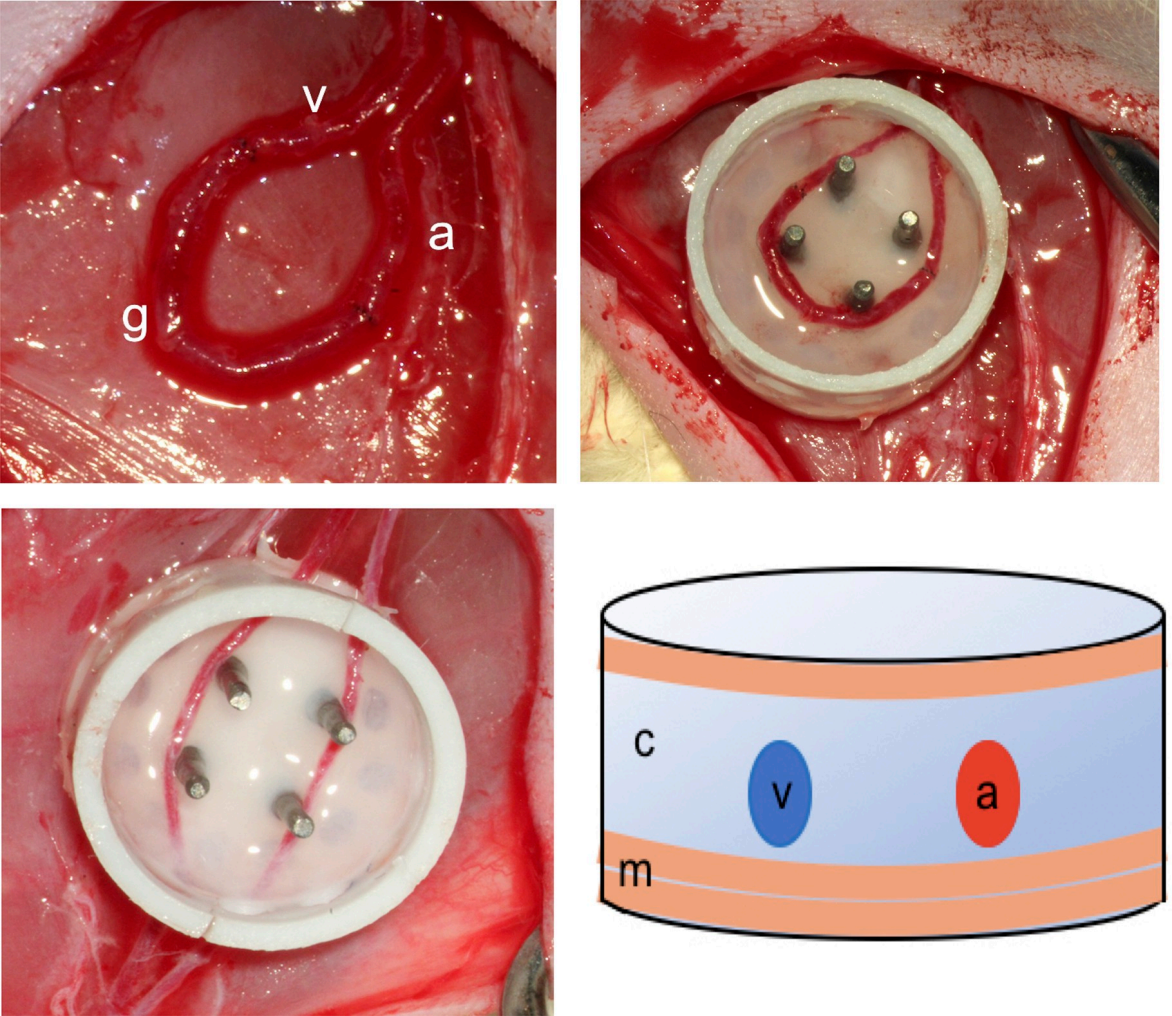
Above left: AV loop: venous graft (g) saphenous artery (a), saphenous vein (v), above right: AV loop in the chamber, below left: AV flow-through vessels passing uninterrupted through the chamber, below right: diagrammatic illustration of a cross section of the construct; a: arterial side of the vascular axis, v: venous side, c: collagen clot, m: Matriderm, 2 layers below and one layer above the vessels.

Custom-made fenestrated teflon chambers were implanted in the groin region (Harhaus Technical and Medical Devices Development and Invention Center, Remscheid, Germany) of 16 Lewis rats. The cylindrical chambers (inner height = 7 mm, inner diameter = 11 mm) are fenestrated to allow additional extrinsic vascularisation after implantation. The constructs were vascularized either through a microsurgically anastomosed arteriovenous loop (AVL group) or through the non-interrupted flow-through saphenous vessels (non-AVL group) (Fig 1). All animals were operated under aseptic conditions by the same surgeon (2^nd^ author). The operations were performed under general inhalational anesthesia (O2-isoflorane 2%, 0.3 ml/min) and all efforts were made to minimize animals’ suffering. To create the AVL, the left saphenous artery and vein were exposed in the subfascial plane. A 2 cm long saphenous venous graft was dissected free from the right thigh and interposed between the saphenous artery and vein of the left side under the operative microscope (Carl Zeiss, Oberkochen, Germany) using 11/0 Polyamid sutures (Ethicon, Norderstadt, Germany). To support the AVL inside the teflon chamber, provide mechanical stability and avoid kinking, two layers of MatriDerm® (MedSkin Solutions, Dr. Suwelack AG, Billerbeck, Germany) were applied on the floor of the chamber before inserting the AVL. After fixing the chamber to the thigh muscles using 6–0 Prolene sutures, 1ml of clotted collagen gel (PureColTM EZ Gel, Sigma-Aldrich, USA) was used to fill the chamber (Fig 1). Before the chamber was closed, another layer of MatriDerm® was applied without any pressure.

To create the non-AVL constructs, the same procedure was applied as in the AVL group except for creating the AVL. Instead of that, the saphenous vessels were passed uninterrupted through a slit in the wall of the chamber after disconnecting their side branches and tributaries. The uninterrupted saphenous vessels represented the flow-through vascular axis inside the construct (Fig 1).

### BMMNC isolation and labeling

Mononuclear cells were isolated from the bone marrow of syngeneic Lewis rats and represented the progenitor cell pool in this study. The BMMNCs isolation and labeling procedures were performed on the same day of cell injection, i.e. 12 days after construct implantation. Under general inhalational anesthesia, syngeneic donor male Lewis rats (age 5–6 weeks) were euthanized by intra-cardiac injection of 1 ml phenobarbital (Narcoren, Boehringer Ingelheim, Ingelheim am Rhein, Germany). The tibia and femur were excised from both legs, and the bone marrow was flushed out with 20 ml bone marrow mesenchymal stem cell (BMSC) culture medium under aseptic conditions. The mononuclear cells were isolated from the bone marrow via density gradient using ficoll medium (Ficoll-PaqueTM plus, GE Healthcare, Sweden) [17]. The cells were then suspended in 1000 μl of BMSC cell culture medium. To determine the cell concentration in the isolate, 1 μl of this cell suspension was mixed with 99 μl of a trypan blue solution (Carl Roth, Karlsruhe, Germany) diluted to 1:100, incubated for three minutes at room temperature and applied to the counting chamber (C-Chip Neubauer, Carl Roth, Karlsruhe, Germany). The vital cells not acquiring the stain were counted within the C-Chip under the microscope to determine the cell concentration in 1μl of the cell isolate. Following the manufacturer protocol, Qdot® 655 nanocrystals (InvitrogenTM / Thermo Fisher Scientific, Germany) were used to label the fresh isolated cells at a concentration of 10 nM [18]. At the beginning of the study, the cell isolation and labeling procedures were repeatedly characterized using FACS analysis. The efficacy of cell labeling, cell viability, erythrocyte/ granulocyte contamination and surface expression of CXCR4, CXCR7, CD45 and CD90 were all verified using the FACSCantoTM II (BD Bioscience, USA) cell sorting analyser. For the FACS analysis, the isolated BMMNCs were first stained with anti-erythrocyte medium (Life Technologies GmbH, Thermo Fisher Scientific, Darmstadt, Germany) and anti-granulocyte labeling (BD Bioscience, USA). This was used to check the efficiency of the ficoll protocol to ensure the sorting out of erythrocytes and granulocytes. In addition, the surface proteins CD45-FITC (BioRad AbD Serotec GmbH, Puchheim, Germany) and C90-PE (BioRad AbD Serotec GmbH, Puchheim, Germany) were analysed. The cells determined as CD45- & CD90 + were considered to represent the mesenchymal stem cells fraction [19]. In a further step, the isolated mononuclear cells were examined for the surface receptors CXCR4 (antibodies-online GmbH, Aachen, Germany), CXCR7 (antibodies-online GmbH, Aachen, Germany).

### Injection of BMMNCs

Twelve days after implantation of the chambers, the isolated and Qdot® labelled BMMNCs were systemically injected either via intravenous (iv) or intra-arterial (ia) route. For the iv injection, 1 × 10^7^ cells were suspended in 1 ml of BMSC cell culture medium and slowly injected via 24G indwelling vein catheter placed in the lateral tail vein. The cannula was then rinsed with 1 ml of normal saline to ensure that all the cells were administered. For the ia injection, a 2 cm long median laparotomy was performed under general inhalational anesthesia. The abdominal aorta was exposed in the retroperitoneal space over a length of 1 cm through blunt dissection. 1 x 10^7^ Qdot®-labelled cells were slowly injected into the abdominal aorta followed by rinsing with 1 ml of saline solution like the iv group. The cannula was removed, and the puncture site compromised for two minutes. The abdominal incision was closed in layers using absorbable 4–0 Vicryl suture (VicrylTM, Ethicon®)

### Explantation of the constructs

Two days after cell injection (14 days after implantation), the animals were sacrificed and the constructs were explanted for analysis. To confirm the patency of the loops during the analysis, the constructs were subjected to India ink perfusion just before sacrifice. Under general inhalational anesthesia, the aorta was cannulated with a 24-gauge cannula, and flushed with 200 ml of warm heparin-Ringer’s solution (100 IU/ml) as a drip infusion. During the procedure, the inferior vena cava was incised to allow exsanguination. Thirty ml of a warm mixture of 15 ml Indian ink (Winsor & Newton, London, UK) and 15 ml gelatin 5%–mannitol 4% solution was then slowly injected into the aorta [20]. The dead animal was then kept in -20°C for 1 hour allowing the mixture to consolidate within the vascular tree. Following that, the implanted constructs, liver, lungs, kidneys, spleen and femoral bone marrow were dissected, fixed in 4% paraformaldehyde solution for 12 hours and then processed for histological analysis. Only animals with patent vascular axes inside the implanted constructs were included in the study for further analysis.

### Cell detection by fluorescence microscopy

**Fig 2:**
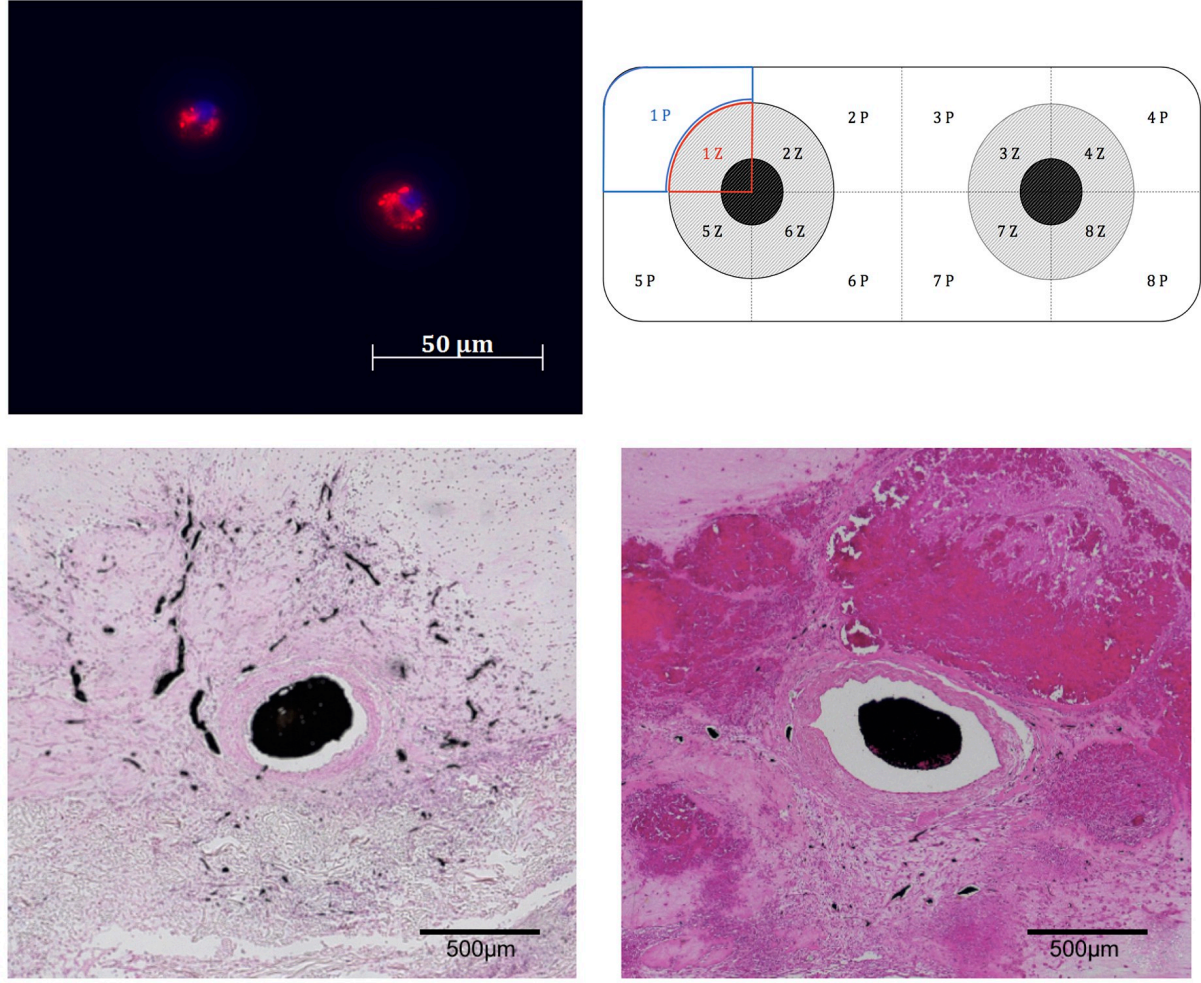
Above left: labelled BMMNCs with 5nM QDots (photo after incubation for 1 week in cell culture showing an intracytoplasmic localization of nanoparticles), above right: diagrammatic illustration of a histological cut section through the construct; P peripheral regions, Z: central regions, below left: HE section of AVL- construct showing angiogenic sprouting as indicated by the india-ink-filled vessels, below right: HE section of the non-AVL construct showing less sprouting out of the vein.

After preparation of standard paraffin blocks, 5 μm-thick sections were obtained from the middle zone of the construct perpendicular to the axis of the AVL (Fig 2). For the initial morphological assessment of the patency of the AVLs, the paraffin sections were stained with hematoxylin-eosin (HE) solution. To detect the distribution of labelled cells, sections from the constructs, lungs, liver, kidneys, spleen and bone marrow were examined under a (Axioimager 2, Carl Zeiss). According to the manufacturer, the Qdot® 655 are excited at a wavelength in the range of 405–615 nm and have a relatively constant emission of 655 nm. The counterstaining of the DNA in the cell nuclei was performed using Hoechst 33342 (Thermo Fisher Scientific, Darmstadt, Germany) and visualized at 350 nm using a DAPI filter. The Qdots appeared brightly fluorescent and aggregated in clumps in the cytoplasm of individual cells. This could also be demonstrated in our preliminary characterization in the cell culture (Fig 2).

### Immunohistological analysis

HIF-1 alpha expression was measured in sections of the explanted constructs using immunofluorescence. The staining was carried out with HIF antibodies at a concentration of 1:200 (HIF-1α antibody, Alexa 546 coupled antibody, sc-13515, Santa Cruz Biotechnology, Inc. Heidelberg, Germany) according to the manufacturer’s protocol. Additionally, the expression of the SDF-1 was analyzed in the constructs using primary antibodies (CXCL12 polyclonal antibody, PA5-89116, InvitrogenTM / Thermo Fisher Scientific, Germany) at a concentration of 1:100 using secondary antibodies (Cy3-AffiniPure Goat Anti-Rabbit IgG (H+L), Jackson ImmunoResearch Laboratories, USA) at a concentration of 1:3000 according to manufacturer’s protocols.

### Histomorphometric evaluation

Histomorphometric evaluation of Qdot® 655 labelled cells was carried out for the liver, spleen and explanted constructs using the Axioimager 2, Carl Zeiss microscope. Regarding the constructs, the labelled cells were counted in 10 sections per chamber. Each section was divided into 8 fields, which were further divided into a peripheral and a central part each (Fig 2). The central zones corresponded to the field of vision of the 100x magnification lens defining the vessel as the center. Regarding the liver and spleen, 8 fields of vision using the 200x magnification lens were randomly chosen and evaluated for the labelled cells. For the comparison between different tissues, the values were presented as a cell density in cells/mm^2^. The histomorphometric evaluation of HIF-1 alpha and SDF-1 expression was done in the constructs using the same scheme. The vascularity of the central regions of the constructs was compared between the AVL and non- AVL groups by counting the India ink filled vessels in the central zones. The tissue growth/cellular infiltration around the vascular axis was similarly evaluated by counting the cells using ImageJ software. The percentage of HIF-1 positive cells in the central regions were calculated in relation to the total cell count and represented the relative central hypoxia.

### RT-PCR analysis

The expression of HIF-1 alpha, SDF-1, CXCR4 and CXCR7 genes was detected in paraffin sections from the constructs using the kit RNeasy FFPE, (QIAGEN GmbH, Hilden, Germany). Relative quantification of messenger ribonucleic acid (mRNA) was performed in a two-step RT- PCR procedure using the universal probe library (Roche, Mannheim, Germany) and a Light Cycler 480 (Roche, Mannheim, Germany) according to the manufacturer’s instructions. The cycling conditions were as follows: 95°C for 5 minutes, 45 cycles consisting of three steps at 95°C for 10seconds, 60°C for 15seconds, and 72°C for 10seconds. Detected mRNA concentrations were done in triplicates and were corrected for 18S ribosomal RNA (rRNA) housekeeping gene in each sample (Table 2). The comparison between various specimens was done using the delta-delta Ct method.

**Table 2:** The studied genes with their sequences and probe number.

Gene	RefSeq	Forward	Reverse	UPL
rCXCR4	NM_022205.3	5’—gCTggAgAgCgAgCATTg-3′	5’—CCTgTTgAAgTTTTCgTTTTCA-3′	38
rCXCR7	NM_053352.1	5’—CAgCACTCAAAgCCAggAA-3′	5’—ggTCTTgAggAgAgCAACCA-3′	26
rHIF1a	NM_024359.1	5’—CATgATggCTCCCTTTTTCA-3′	5’—ACATAgTAggggCACggTCA-3′	18
rSDF1	NM_022177.3	5’—CCCTgCCgATTCTTTgAg-3′	5’—gCTTTTCAgCCTTgCAACA-3′	21

### Statistical analysis

The count of detected Qdot® 655 labelled cells, central blood vessels, central cellular infiltration, HIF-1alpha, and SDF-1 positive cells in all counted fields and sections was presented in the form of the arithmetic mean and the standard deviation. The comparison between various groups (AVL vs non-AVL, intra-arterial vs. intravenous injection) was done via the unpaired t-test using the GraphPad Prism 8 software for Mac. For all performed statistical tests, p-values <0.05 were considered as statistically significant.

## Results

### Surgical procedures

All animals tolerated the surgical and cell injection procedures. Neither thrombotic nor allergic reactions were documented after cell injection. No wound complications were noticed during the 2 weeks-follow up period.

### BMMNC isolation, Qdot® 655 labelling and FACS analysis

Cell isolation using the Ficoll-protocol rendered an average mononuclear cell count of 4.97 × 10^7^ ± 1.3 × 10^6^ cells. The cell viability detected via trypan blue staining was more than 95%. The FACS analysis showed an average erythrocyte and granulocyte contamination of 3.08% ± 0.86% and 8.58% ± 2.42% respectively. The CD45- & CD90 + cells represented an average quote of 0.60 ± 0.12%. Detection of the chemokine receptors showed that 4.97% ± 0.95% of the cells expressed CXCR4 receptor, 10.54% ± 4.97% expressed CXCR7 receptor, and 5.23% ± 1.63% expressed both receptors. After performing the Qdot® 655 cell labeling, 83.36% ± 2.37% of the cells showed a positive signal as shown by the FACS analysis. The intracellular Qdots exhibited a broad excitation range between 405–615 nm and a narrow emission width with a peak at 655 nm. A qualitative evaluation of labelled cells in the cell culture using the fluoresence microscope (Axioimager 2, Carl Zeiss) confirmed the intracytoplasmic distribution of the Qdots (Fig 2).

### Distribution of Qdot® 655 cells after injection

**Fig 3:**
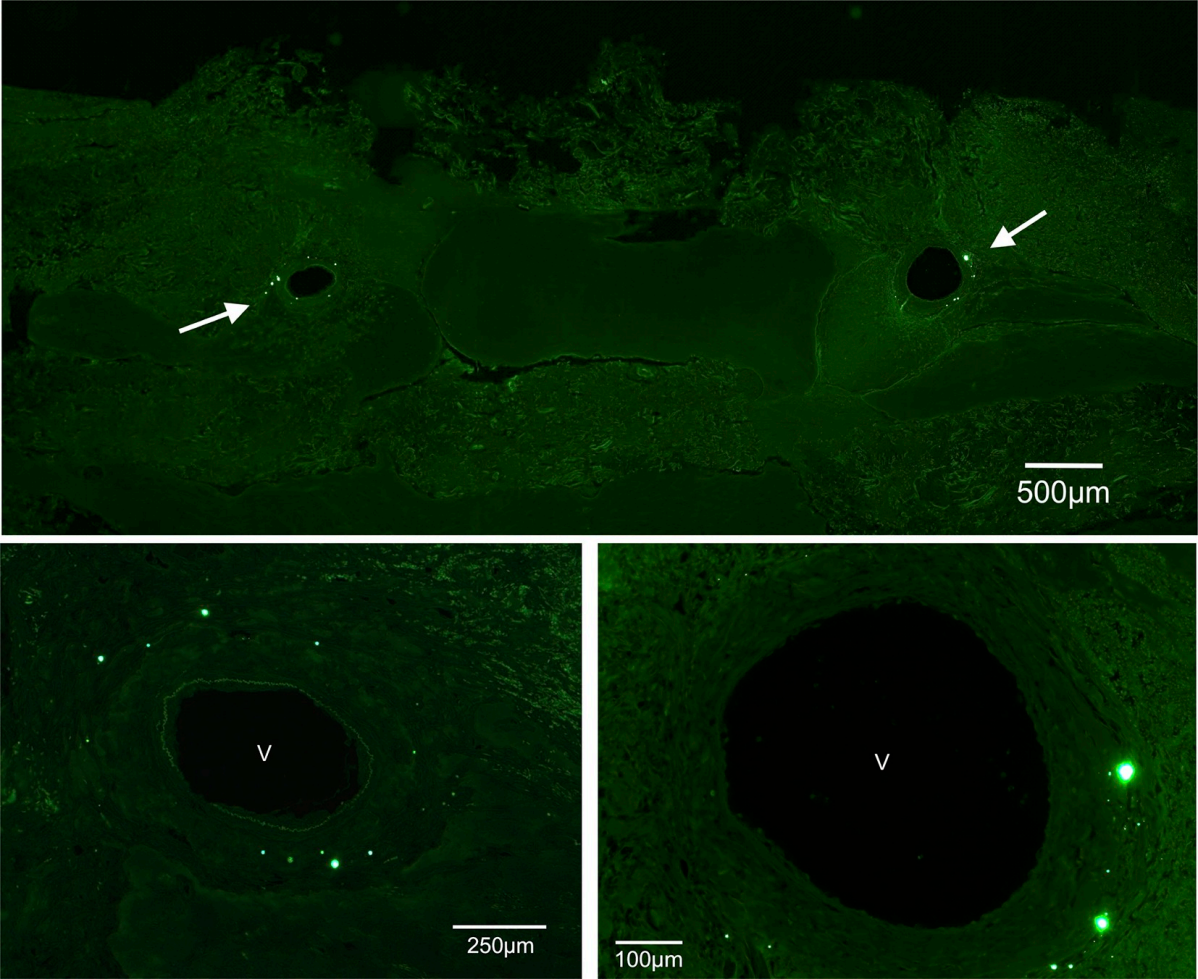
Fluorescence microscopy; above: overview of a cut section through an AVL-construct showing QDots around the vessels (arrows). Below: higher magnification showing the labelled cells in clusters leading to an intensive signaling, v: cross section of the vessel, green filter, peak emission at 565 nm.

**Fig 4:**
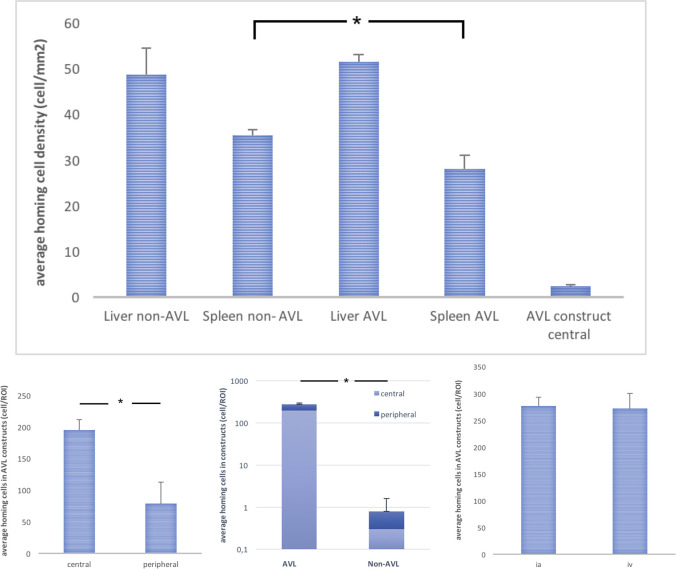
Histomorphometric analysis of cell engraftment in various tissues 48h after injection as detected by fluorescence microscopy, (*) indicates statistical significance.

At the defined time point (2 days after cell injection), no cells could be detected in the tissues of the lungs, kidneys or bone marrow. This was observed in all groups both after intra-arterial and intravenous injections. The Qdot® 655 signals were detected exclusively in the liver, spleen, AVL constructs and to a minimal extent in the non-AVL constructs. In the liver and spleen, the Qdots showed a peak emission at 655nm. Within the constructs, the Qdot signals exhibited a green shift with a peak emission at 565 nm. A significant difference could be detected between the number of labelled cells in the AVL and non-AVL constructs with much more cells detected in the AVL constructs both in central and peripheral zones (*p* <0.0001). In the AVL constructs, the cells were mainly detected at the central zones around the AVL, significantly more than those in peripheral zones (*p*<0.0001) (Fig 3). Regarding the tissues of the reticuloendothelial system, significantly more cells could be detected in the liver than the spleen (*p* <0.0001). Significantly less labelled cells were found in the spleen of the AVL constructs than in the spleen of the non- AVL constructs (*p* < 0.0001) (Fig 4). No differences could be detected between ia and iv injection methods among all groups. The detected labelled cell and their corresponding densities are shown in Table 3.

**Table 3:** Detected labelled cells among different tissues; cell/ROI (cell/mm^2^).

	Liver	Spleen	Construct	
			central	peripheral
AVL group	427.7±13.7 (51.5±1.6)	232.7±25.1 (28±3)	195.3±16.6 (2.4±0.2)	79.3±33.2
non-AVL group	403.7±48.2 (48.6±5.8)	293.6±9.7 (35.3±1.2)	0.3±0.5 (0.004±0.006)	0.5±0.7
*p*- value	0.3	< 0.0001	< 0.0001	< 0.0001

### Histology and immunofluorescence

**Fig 5:**
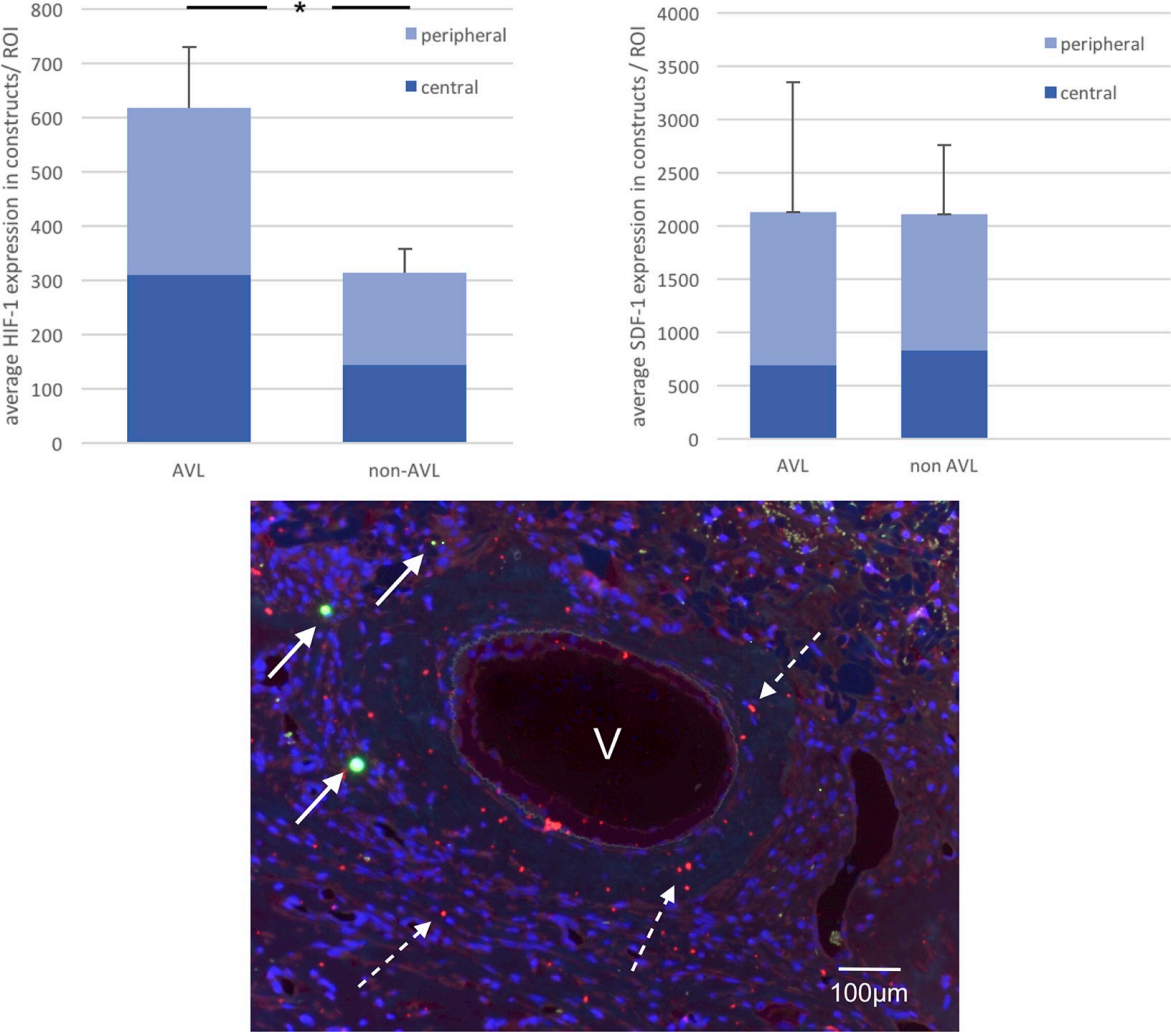
Above: histomorphometric analysis of HIF-1 and SDF-1 expression within the constructs, (*) indicates statistical significance. Below: combined Immunofluorescence image of AVL construct showing the vascular axis (v), the homing cells labelled with QDots in green (arrows), the SDF-1 expression in red around the vessel (dotted arrows) and the cell nuclei in blue stained with Hoechst.

**Fig 6:**
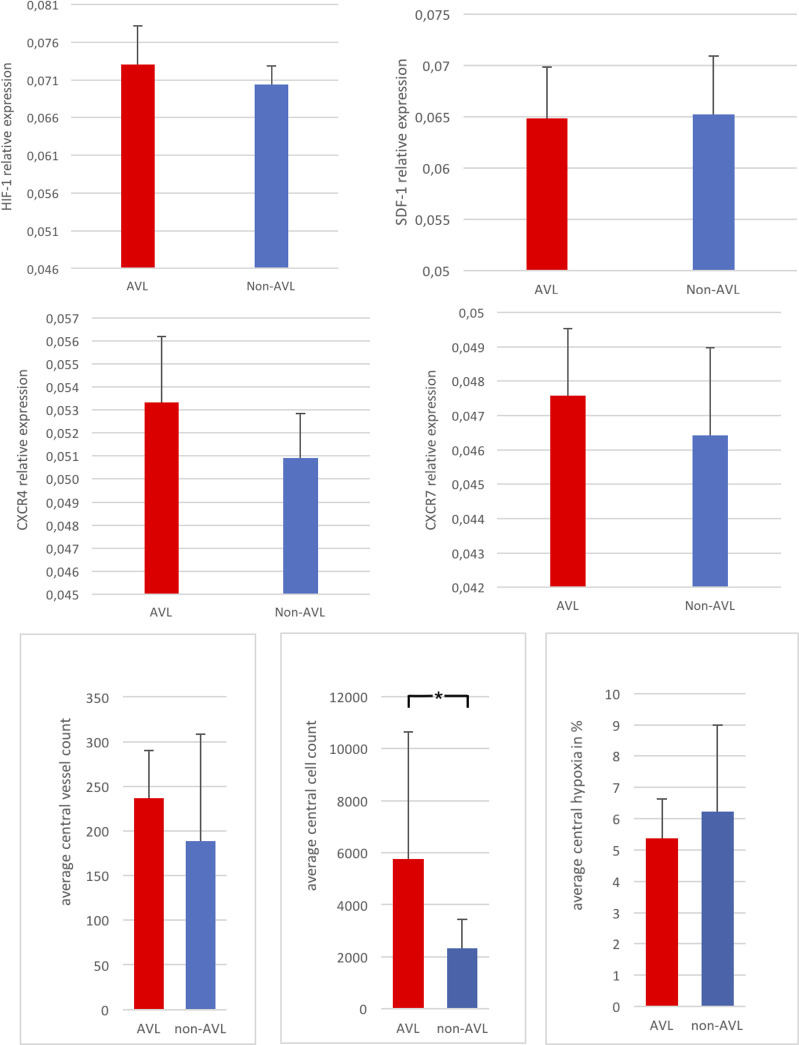
Above: RT-PCR analysis of relative expression of HIF-1, SDF-1, CXCR4, and CXCR7 in the constructs, below: histomorphometric analysis of central vessel count, central total cell count and central relative hypoxia (HIF-1 positive cells in relation to total cell count), (*) indicates statistical significance.

No histological signs of embolization or immune reaction to the injected cells were detected in the tissues of the spleen, liver, lungs, kidneys or bone marrow. The qualitative analysis of the HE stained sections confirmed the patency of the vascular axes in the AVL constructs. The lumen of the AVL was filled with India ink and was surrounded by a dense vascularization and new tissue formation (Fig 2). Extrinsic vascularization through the fenestrations of the chamber was also detected in all constructs. The central vascularity within the AVL constructs was higher than that of the non-AVL. The detected difference was however not statistically significant (*p* = 0.54). The cellular growth/cellular infiltration around the vascular axes was significantly more within the AVL constructs (*p* = 0.046). The histomorphometric analysis also showed a significant increase in HIF-1 absolute expression in the AVL group in comparison to the non-AVL group both in central and peripheral zones of the constructs (*p*<0,0001, Fig 5). Within individual constructs, however, no difference could be seen between HIF-1 expression in the central and peripheral zones. The relative central hypoxia (calculated via the percentage of HIF-1 positive cells in relation to the whole cell count in central zones) was lower within the AVL constructs in comparison to the non-AVL constructs, but the difference was not statistically significant (Fig 6, *p* = 0.5).

Regarding SDF-1, no significant differences could be detected between AVL and non-AVL constructs. Within the individual constructs the expression of SDF-1 was peripherally higher in both AVL und non-AVL groups. This was even statistically significant in the non-AVL constructs (*p* = 0.049).

### Real time PCR

All the tested genes could be adequately detected among all groups. The average expression of HIF-1 in the AVL constructs showed a 1.4-fold increase than that of the non-AVL constructs. However, SDF-1 expression was similar in both groups. Regarding the chemokine receptor expression in the AVL group, CXCR4 showed a 1.8-fold increase in expression than that of the non-AVL constructs. Similarly, the expression of CXCR7 was 1.5-fold higher in the AVL group (Fig 6).

## Discussion

To the best of our knowledge, this study shows for the first time that tissue engineering constructs axially vascularized via AV loops can specifically attract systemically injected bone marrow mononuclear cells without an additional extrinsic chemokine stimulation. The distribution of the labelled cells around the central vascular axis indicates that the cells reached the constructs via the AV loop, as this effect could not be detected at the peripheral zones or in similar constructs vascularized via flow-through vessels at the studied time point. This capability to attract BMMNCs from the systemic circulation is not only important to understand the mechanism of vascularisation and tissue formation induced by the AVL, but is also of outmost relevance to cell therapy strategies especially regarding the optimization of cell engraftment to target tissues or organs.

We compared in this study the ability of axially vascularized constructs either via AV loops or flow-through vessels to attract injected BMMNCs. AV loops, AV bundles and flow-through vessels are well established methods for axial vascularization, which is now considered a corner stone for tissue engineering of clinically relevant 3D tissues [1, 21]. The AV loops are known to have superior angiogenic and tissue growth potentials compared to other modalities [22], which is probably related to the initial enhanced blood flow [8] and to the state of relative hypoxia within the construct [9]. In our study, the AVL group should superior results regarding the central vascularity and a statistically significant enhanced tissue growth.

We injected the cells at day 12 after construct implantation. This time point was based upon our previous experience with the hemodynamics of the AVL. Before 12 days, the vessel sprouting from the AV loop is minimal [23], and therefore an adequate vascular bed would not be available inside the chamber. Furthermore, the hemodynamics of the AVL at earlier time points are those of an arteriovenous fistula with a substantially higher blood flow [8]. Applying the cells under these conditions would cause a bias regarding cell engraftment in comparison to the control group especially after ia injection.

We did not notice a difference between intra-arterial and intravenous injection methods regarding the engraftment of the labelled cells 2 days after injection. The intravenously injected cells were probably not captured in the lung vasculature denoting an irrelevant pulmonary first pass effect. This could be related to the different adhesion abilities and to the small size of the BMMNCs (cell diameter about 7 μm) in comparison to other progenitor cell types as mesenchymal stem cells or *in vitro* cultivated cells [14, 24, 25]. Another explanation would be that a fraction of the cells was initially trapped in the lungs after iv injection and slowly mobilized out of the lung over 2 days. This ‘‘temporary” entrapment in lung tissue was previously described in several studies [26, 27]. The enhanced engraftment of progenitor cells within the AVL constructs even after intravenous injection may introduce these constructs as a safe and practical method to enhance cell engraftment to target tissues during cell therapies.

Two days after injection, the cells were detected mainly in the liver, spleen and to a lesser extent in the AVL constructs. No cells could be detected in other examined tissues of the kidneys or lungs denoting that their homing was related to a chemotactic signaling rather than a mere hemodynamic distribution, a conclusion reinforced by the similar distribution following ia and iv injections. The splenic engraftment in the AVL group was significantly less than that of the non-AVL group. This may denote that a cell mobilization took place primarily from the spleen reservoir. Probably the cells resided initially in the reticuloendothelial system (mainly liver and spleen) after injection and were then chemotactically mobilized towards the AVL constructs over two days. This conclusion, however, should be verified through further cell tracking studies including assessment of cell engraftment at earlier time points.

The homing cell densities in the liver and spleen were substantially higher than the central AVL construct zones (28–51.5 vs 2.4/mm^2^). This finding, however, is a snapshot calculation at the studied time point (2 days after injection) and does not represent the final engraftment density within the constructs. It should also be noted that the calculated number of homing cells in the AVL constructs are only that of the labelled systemically injected cells. We assume, however, that the same scenario applies to the non-labelled endogenous BMMNCs of the animal, which were probably mobilized and competed with the labelled cells for the same receptor sites inside the AVL constructs. This means in turn that the whole number of progenitor cells homing to the AVL constructs could be probably higher than that of the applied/labelled cells.

We investigated the underlying mechanism for this differential progenitor cell homing focusing on the role of hypoxia and the SDF1-CXCR4-7 axis. We noticed a significantly higher absolute expression of HIF-1 in the AVL constructs in comparison to the non-AVL group. However, and due to the robust cellular infiltration and tissue growth around the AVL, the relative HIF-1 expression in relation to the amount of tissue growth was lower in comparison to the non-AVL constructs. The enhanced absolute expression was in the context of a controlled hypoxia, as there was no histological evidence of tissue necrosis in any of the constructs. These findings may denote a correlation between this state of controlled hypoxia inside the AVL constructs and their ability to attract the labelled cells. An enhanced HIF-1 expression was previously evidenced in AVL constructs and was suggested as one of the factors responsible for angiogenesis and tissue formation in the AVL model [9, 28, 29]. In our previous experience with AVL constructs after irradiation, we detected an enhanced bone differentiation after high dose irradiation, which could not be attributed to the *in-situ* stem cell population which was surely damaged by irradiation. We hypothesized at that time that the enhanced hypoxia after irradiation led to chemotaxis of endogenous progenitor cells and consequently enhanced bone formation [10, 30]. The current results support our previous hypothesis as they have shown an enhanced progenitor cell engraftment related to an enhanced state of hypoxia, which had probably been augmented by the irradiation in our previous experiments.

In the current study a state of homogenous controlled hypoxia was present within the construct. We could not detect a significant difference between HIF-1 expression in the central and peripheral zones of the constructs, most probably due to the fenestration of our chamber which allowed extrinsic vascularization in addition to the intrinsic vascularization via the AVL. In other models of the AVL chamber without fenestration, a clear HIF-1 gradient could be demonstrated with more expression towards the periphery [9]. Despite the homogenous HIF-1 expression in our constructs, the labelled BMMNCs were detected mainly around the AVL and not at the periphery, denoting that the AVL was the main access of the progenitor cells to the construct despite the fenestrations in the chamber. This emphasizes the uniqueness of the AVL model in possessing a hypoxic stimulus hand and in hand with an adequate access to the progenitor cell niche, a model that resembles to great extent the model of embryogenesis [31].

The definite mechanism of cell homing is not yet clear. A suggested explanation is an enhanced HIF-1—SDF-1 cascade leading to progenitor cell attraction via surface receptors CXCR4 and CXCR7 [15, 16]. Our RT-PCR results confirmed an enhanced expression of CXCR4 and CXCR7 in the AVL constructs which indicates an enhanced engraftment of cells expressing these receptors including, but not limited to, the labelled BMMNCs. However, and quite contrary to HIF-1, there was no difference in SDF-1 expression between AVL and non-AVL groups. This means that the CXCR4 and CXCR7 positive cells were probably recruited via a mechanism independent on SDF-1 expression inside the AVL constructs. Similarly, Simcock et al. could show an enhanced engraftment of systemically injected endothelial cell precursors (EPCs) to an AVL chamber only after local application of exogenous SDF-1 (CXCL12) into the chambers [32]. Our observations suggest that at the studied time point and under normal conditions, the chemotaxis of progenitor cells towards the AVL constructs occurs via a HIF-1 dependent but non-SDF-1 dependent mechanism. Probably, the monocytes/macrophages fractions of BMMNCs were the first to reach the AVL constructs induced by a direct stimulus from the HIF-1 and without SDF-1 stimulation. Their activation will further lead to secretion of monocyte chemoattractant protein 1 (MCP-1), which recruits more monocytes and further induces HIF-1 [33, 34]. This is also supported by the previously evidenced double staining of HIF-1 and ectodysplasin-1 in the AVL constructs between 7–14 days indicating that macrophages accounted for a significant fraction of the cells at this phase [9]. In a further step, HIF-1 will lead to activation of SDF-1 and VEGF inducing each other in a positive feedback loop. An overexpression of SDF-1 will probably occur at a later time point and will provoke the known chemotactic effects on other progenitor and stem cells [15, 16, 35].

We labeled the cells with Qtracker® nanocrystals to assure a continuous illumination without the degradation problems often associated with organic dyes [36]. Qtracker® 655 labels are distributed in vesicles in the cytoplasm, excited at a wavelength in the range of 405–615 nm and have an emission of 655 nm. In the liver and spleen, the Qdots showed a typical red peak emission at 655nm. The Qdots signals in the constructs however, showed a green shift with a typical peak emission at 565 nm. Furthermore, the nanocrystals in the constructs appeared more in clusters leading to an intensive signaling (Fig 3). This green shift could be attributed to a degradation of the PEG (polyethylene glycol) coating of the nanocrystals which could occur in acidic conditions (Dr. Gebhardt, Thermo Fisher Scientific, Germany, personal communication, February 25, 2020). Within pH values below 4, even the metallic core of the nanocrystals may start to degrade. This leads to decreasing the size of the crystals and consequently changing the emission range towards green [37]. Other studies showed that Qdots were first sequestered in early endosomes, which were slowly acidified and turned into late endosomes and lysosomes. Their distribution was shown also to be more densely packed in late endosomes/lysosomes, presumably due to the acidic pH therein [38]. This phenomenon was clearly seen in the constructs mostly due to the acidic conditions within the constructs triggered by hypoxia, degradation of the collagen inside the chamber into amino acids and the predominance of macrophages with their prominent lysosomal activity. Another issue would be the possible effect of Qdots on cell maturation and surface marker expression such as CXCR4 and 7 especially on long term. This effect was mentioned in literature in different cell lines with some contradictory results [39, 40]. We believe, however, that such an effect would be minimal according to our study design as we injected the cells immediately after cell labeling.

Our current findings provide insight into the underlying mechanism of vascularisation and tissue formation via AVLs, proving a definite role of enhanced cell recruitment from the systemic circulation. This knowledge may be beneficial in applying this well-established microsurgical model in cell therapies, where directing the injected cells to the target organs remains a major challenge [14]. Furthermore, a detailed characterization of the chemotactic potential of the AVL constructs could help enrich the tissue engineering constructs via systemic injection of cells. This issue remains a major concern in tissue engineering because the local application of cells to a well vascularized scaffold at an optimal time point is technically difficult.

Another important issue is the theoretical ability of the AVL constructs to attract cancer stem cells leading to cancer recurrence if AVL constructs were used for reconstruction after cancer ablation. There is growing evidence that recurrence after radiation therapy (for example of brain tumors) is related to enhanced tissue hypoxia, HIF-1 overexpression and consequently SDF-1 expression leading to infiltrating macrophages, which may subsequently be responsible for radiation-induced tumor invasiveness [41]. Other studies highlighted the role of hypoxia on the maintenance and evolution of cancer stem cells [42]. From a clinical point of view, however, the induced hypoxia in the AVL construct is nothing more than a temporary tissue hypoxia which sometimes follows pedicled or free flap reconstruction commonly done after cancer ablation.

The study has some limitations that would be optimized in further research. Studying the hypoxia profile and consequently the chemotactic potential at other time points including long-term-dynamics of cell distribution would be of outmost importance to characterize the model and further solidify the current findings. Further investigations to uncover the mechanism of chemotaxis including the role of MCP-1 together with more investigations on the SDF-1—CXCR4 and CXCR7 axis would be necessary. Finally, the cells homing to the AVL constructs should be characterized further to find out which fraction of BMMNCs pool plays the main role in the homing process.

## Supporting information

S1 Dataset Raw analysis data. (XLSX)

S1 File Detailed materials and methods. (DOCX)

## References

[1] Sparks DS, Savi FM, Saifzadeh S, Schuetz MA, Wagels M, Hutmacher DW. Convergence of Scaffold-Guided Bone Reconstruction and Surgical Vascularization Strategies-A Quest for Regenerative Matching Axial Vascularization. Front Bioeng Biotechnol. 2019;7:448. Epub 2020/01/31. doi: 10.3389/fbioe.2019.00448 ; PubMed Central PMCID: PMC6967032. 31998712 PMC6967032

[2] Weigand A, Horch RE, Boos AM, Beier JP, Arkudas A. The Arteriovenous Loop: Engineering of Axially Vascularized Tissue. Eur Surg Res. 2018;59(3–4):286–99. Epub 2018/09/24. doi: 10.1159/000492417 . 30244238

[3] Erol OO, Spira M. New capillary bed formation with a surgically constructed arteriovenous fistula. Surg Forum. 1979;30:530–1. Epub 1979/01/01. 395707. 395707

[4] Lokmic Z, Stillaert F, Morrison WA, Thompson EW, Mitchell GM. An arteriovenous loop in a protected space generates a permanent, highly vascular, tissue-engineered construct. FASEB J. 2007;21(2):511–22. Epub 2006/12/19. doi: 10.1096/fj.06-6614com . 17172640

[5] Horch RE, Beier JP, Kneser U, Arkudas A. Successful human long-term application of in situ bone tissue engineering. J Cell Mol Med. 2014;18(7):1478–85. Epub 2014/05/08. doi: 10.1111/jcmm.12296 ; PubMed Central PMCID: PMC4124030. 24801710 PMC4124030

[6] Eweida AM, Horch RE, Marei MK, Elhammady HA, Etaby AN, Nabawi AS, et al. Axially vascularised mandibular constructs: Is it time for a clinical trial? J Craniomaxillofac Surg. 2015;43(7):1028–32. Epub 2015/05/11. doi: 10.1016/j.jcms.2014.10.018 . 25958095

[7] Eweida A. Mandibular Reconstruction Using Tissue Regeneration With Axially Vascularised Bone Substitutes. Alexandria, Egypt 2019.

[8] Schmidt VJ, Hilgert JG, Covi JM, Leibig N, Wietbrock JO, Arkudas A, et al. Flow increase is decisive to initiate angiogenesis in veins exposed to altered hemodynamics. PLoS One. 2015;10(1):e0117407. Epub 2015/01/31. doi: 10.1371/journal.pone.0117407 ; PubMed Central PMCID: PMC4312013. 25635764 PMC4312013

[9] Yuan Q, Arkudas A, Horch RE, Hammon M, Bleiziffer O, Uder M, et al. Vascularization of the Arteriovenous Loop in a Rat Isolation Chamber Model-Quantification of Hypoxia and Evaluation of Its Effects. Tissue Eng Part A. 2018;24(9–10):719–28. Epub 2017/10/06. doi: 10.1089/ten.TEA.2017.0262 . 28978278

[10] Eweida A, Frisch O, Giordano FA, Fleckenstein J, Wenz F, Brockmann MA, et al. Axially vascularized tissue-engineered bone constructs retain their in vivo angiogenic and osteogenic capacity after high-dose irradiation. J Tissue Eng Regen Med. 2018;12(2):e657–e68. Epub 2016/10/04. doi: 10.1002/term.2336 . 27696709

[11] Wang C, Li J, Zhang B, Li Y. Safety and efficacy of bone marrow-derived cells therapy on cardiomyopathy: a meta-analysis. Stem Cell Res Ther. 2019;10(1):137. Epub 2019/05/22. doi: 10.1186/s13287-019-1238-5 ; PubMed Central PMCID: PMC6528271. 31109372 PMC6528271

[12] Rennert RC, Sorkin M, Garg RK, Gurtner GC. Stem cell recruitment after injury: lessons for regenerative medicine. Regen Med. 2012;7(6):833–50. Epub 2012/11/21. doi: 10.2217/rme.12.82 ; PubMed Central PMCID: PMC3568672. 23164083 PMC3568672

[13] Liao GP, Harting MT, Hetz RA, Walker PA, Shah SK, Corkins CJ, et al. Autologous bone marrow mononuclear cells reduce therapeutic intensity for severe traumatic brain injury in children. Pediatr Crit Care Med. 2015;16(3):245–55. Epub 2015/01/13. doi: 10.1097/PCC.0000000000000324 ; PubMed Central PMCID: PMC4351120. 25581630 PMC4351120

[14] Kean TJ, Lin P, Caplan AI, Dennis JE. MSCs: Delivery Routes and Engraftment, Cell-Targeting Strategies, and Immune Modulation. Stem Cells Int. 2013;2013:732742. Epub 2013/09/04. doi: 10.1155/2013/732742 ; PubMed Central PMCID: PMC3755386. 24000286 PMC3755386

[15] Ceradini DJ, Kulkarni AR, Callaghan MJ, Tepper OM, Bastidas N, Kleinman ME, et al. Progenitor cell trafficking is regulated by hypoxic gradients through HIF-1 induction of SDF-1. Nat Med. 2004;10(8):858–64. Epub 2004/07/06. doi: 10.1038/nm1075 . 15235597

[16] Murphy PM, Heusinkveld L. Multisystem multitasking by CXCL12 and its receptors CXCR4 and ACKR3. Cytokine. 2018;109:2–10. Epub 2018/02/06. doi: 10.1016/j.cyto.2017.12.022 ; PubMed Central PMCID: PMC6003845. 29398278 PMC6003845

[17] Boyum A. Isolation of mononuclear cells and granulocytes from human blood. Isolation of monuclear cells by one centrifugation, and of granulocytes by combining centrifugation and sedimentation at 1 g. Scand J Clin Lab Invest Suppl. 1968;97:77–89. Epub 1968/01/01. . 4179068

[18] Muller-Borer BJ, Collins MC, Gunst PR, Cascio WE, Kypson AP. Quantum dot labeling of mesenchymal stem cells. J Nanobiotechnology. 2007;5:9. Epub 2007/11/09. doi: 10.1186/1477-3155-5-9 ; PubMed Central PMCID: PMC2186355. 17988386 PMC2186355

[19] Boxall SA, Jones E. Markers for characterization of bone marrow multipotential stromal cells. Stem Cells Int. 2012;2012:975871. Epub 2012/06/06. doi: 10.1155/2012/975871 ; PubMed Central PMCID: PMC3361338. 22666272 PMC3361338

[20] Kneser U, Polykandriotis E, Ohnolz J, Heidner K, Grabinger L, Euler S, et al. Engineering of vascularized transplantable bone tissues: induction of axial vascularization in an osteoconductive matrix using an arteriovenous loop. Tissue Eng. 2006;12(7):1721–31. Epub 2006/08/08. doi: 10.1089/ten.2006.12.1721 . 16889503

[21] Eweida AM, Nabawi AS, Elhammady HA, Marei MK, Khalil MR, Shawky MS, et al. Axially vascularized bone substitutes: a systematic review of literature and presentation of a novel model. Arch Orthop Trauma Surg. 2012;132(9):1353–62. Epub 2012/05/31. doi: 10.1007/s00402-012-1550-3 . 22643804

[22] Tanaka Y, Sung KC, Tsutsumi A, Ohba S, Ueda K, Morrison WA. Tissue engineering skin flaps: which vascular carrier, arteriovenous shunt loop or arteriovenous bundle, has more potential for angiogenesis and tissue generation? Plast Reconstr Surg. 2003;112(6):1636–44. Epub 2003/10/28. doi: 10.1097/01.PRS.0000086140.49022.AB . 14578795

[23] Polykandriotis E, Tjiawi J, Euler S, Arkudas A, Hess A, Brune K, et al. The venous graft as an effector of early angiogenesis in a fibrin matrix. Microvasc Res. 2008;75(1):25–33. Epub 2007/06/05. doi: 10.1016/j.mvr.2007.04.003 . 17544455

[24] Fischer UM, Harting MT, Jimenez F, Monzon-Posadas WO, Xue H, Savitz SI, et al. Pulmonary passage is a major obstacle for intravenous stem cell delivery: the pulmonary first-pass effect. Stem Cells Dev. 2009;18(5):683–92. Epub 2008/12/23. doi: 10.1089/scd.2008.0253 ; PubMed Central PMCID: PMC3190292. 19099374 PMC3190292

[25] Kyriakou C, Rabin N, Pizzey A, Nathwani A, Yong K. Factors that influence short-term homing of human bone marrow-derived mesenchymal stem cells in a xenogeneic animal model. Haematologica. 2008;93(10):1457–65. Epub 2008/08/30. doi: 10.3324/haematol.12553 . 18728032

[26] Gao J, Dennis JE, Muzic RF, Lundberg M, Caplan AI. The dynamic in vivo distribution of bone marrow-derived mesenchymal stem cells after infusion. Cells Tissues Organs. 2001;169(1):12–20. Epub 2001/05/08. doi: 10.1159/000047856 . 11340257

[27] Kang WJ, Kang HJ, Kim HS, Chung JK, Lee MC, Lee DS. Tissue distribution of 18F-FDG-labeled peripheral hematopoietic stem cells after intracoronary administration in patients with myocardial infarction. J Nucl Med. 2006;47(8):1295–301. Epub 2006/08/03. . 16883008

[28] Hofer SO, Mitchell GM, Penington AJ, Morrison WA, RomeoMeeuw R, Keramidaris E, et al. The use of pimonidazole to characterise hypoxia in the internal environment of an in vivo tissue engineering chamber. Br J Plast Surg. 2005;58(8):1104–14. Epub 2005/07/27. doi: 10.1016/j.bjps.2005.04.033 . 16043148

[29] Yuan Q, Bleiziffer O, Boos AM, Sun J, Brandl A, Beier JP, et al. PHDs inhibitor DMOG promotes the vascularization process in the AV loop by HIF-1a up-regulation and the preliminary discussion on its kinetics in rat. BMC Biotechnol. 2014;14:112. Epub 2014/12/30. doi: 10.1186/s12896-014-0112-x ; PubMed Central PMCID: PMC4298964. 25543909 PMC4298964

[30] Eweida A, Fathi I, Eltawila AM, Elsherif AM, Elkerm Y, Harhaus L, et al. Pattern of Bone Generation after Irradiation in Vascularized Tissue Engineered Constructs. J Reconstr Microsurg. 2018;34(2):130–7. Epub 2017/10/31. doi: 10.1055/s-0037-1607322 . 29084413

[31] Fathollahipour S, Patil PS, Leipzig ND. Oxygen Regulation in Development: Lessons from Embryogenesis towards Tissue Engineering. Cells Tissues Organs. 2018;205(5–6):350–71. Epub 2018/10/03. doi: 10.1159/000493162 ; PubMed Central PMCID: PMC6397050. 30273927 PMC6397050

[32] Simcock JW, Penington AJ, Morrison WA, Thompson EW, Mitchell GM. Endothelial precursor cells home to a vascularized tissue engineering chamber by application of the angiogenic chemokine CXCL12. Tissue Eng Part A. 2009;15(3):655–64. Epub 2008/12/09. doi: 10.1089/ten.tea.2007.0438 . 19061426

[33] Murdoch C, Giannoudis A, Lewis CE. Mechanisms regulating the recruitment of macrophages into hypoxic areas of tumors and other ischemic tissues. Blood. 2004;104(8):2224–34. Epub 2004/07/03. doi: 10.1182/blood-2004-03-1109 . 15231578

[34] Schatteman GC, Dunnwald M, Jiao C. Biology of bone marrow-derived endothelial cell precursors. Am J Physiol Heart Circ Physiol. 2007;292(1):H1–18. Epub 2006/09/19. doi: 10.1152/ajpheart.00662.2006 . 16980351

[35] Youn SW, Lee SW, Lee J, Jeong HK, Suh JW, Yoon CH, et al. COMP-Ang1 stimulates HIF-1alpha-mediated SDF-1 overexpression and recovers ischemic injury through BM-derived progenitor cell recruitment. Blood. 2011;117(16):4376–86. Epub 2011/01/05. doi: 10.1182/blood-2010-07-295964 . 21200018

[36] Ballou B, Lagerholm BC, Ernst LA, Bruchez MP, Waggoner AS. Noninvasive imaging of quantum dots in mice. Bioconjug Chem. 2004;15(1):79–86. Epub 2004/01/22. doi: 10.1021/bc034153y . 14733586

[37] Domingos RF, Franco C, Pinheiro JP. Stability of core/shell quantum dots—role of pH and small organic ligands. Environ Sci Pollut Res Int. 2013;20(7):4872–80. Epub 2013/01/12. doi: 10.1007/s11356-012-1457-0 . 23307080

[38] Xiao Y, Forry SP, Gao X, Holbrook RD, Telford WG, Tona A. Dynamics and mechanisms of quantum dot nanoparticle cellular uptake. J Nanobiotechnology. 2010;8:13. Epub 2010/06/17. doi: 10.1186/1477-3155-8-13 ; PubMed Central PMCID: PMC2898766. 20550705 PMC2898766

[39] Steponkiene S, Kavaliauskiene S, Purviniene R, Rotomskis R, Juzenas P. Quantum dots affect expression of CD133 surface antigen in melanoma cells. Int J Nanomedicine. 2011;6:2437–44. Epub 2011/11/11. doi: 10.2147/IJN.S24477 ; PubMed Central PMCID: PMC3205138. 22072879 PMC3205138

[40] Rak-Raszewska A, Marcello M, Kenny S, Edgar D, See V, Murray P. Quantum dots do not affect the behaviour of mouse embryonic stem cells and kidney stem cells and are suitable for short-term tracking. PLoS One. 2012;7(3):e32650. Epub 2012/03/10. doi: 10.1371/journal.pone.0032650 ; PubMed Central PMCID: PMC3293847. 22403689 PMC3293847

[41] Wang SC, Yu CF, Hong JH, Tsai CS, Chiang CS. Radiation therapy-induced tumor invasiveness is associated with SDF-1-regulated macrophage mobilization and vasculogenesis. PLoS One. 2013;8(8):e69182. Epub 2013/08/14. doi: 10.1371/journal.pone.0069182 ; PubMed Central PMCID: PMC3734136. 23940516 PMC3734136

[42] Tong WW, Tong GH, Liu Y. Cancer stem cells and hypoxia-inducible factors (Review). Int J Oncol. 2018;53(2):469–76. Epub 2018/05/31. doi: 10.3892/ijo.2018.4417 . 29845228

