# Supplementary material for: Systemically injected bone marrow mononuclear cells specifically home to axially vascularized tissue engineering constructs
Source: PLoS One. 2022 Aug 11;17(8):e0272697. doi: 10.1371/journal.pone.0272697 (PMC9371259; doi:10.1371/journal.pone.0272697)
Supplement: S1 File — (DOCX) [file pone.0272697.s002.docx]

**Materials and Methids**

**1. Standardization of rBMMNC isolation**

**1.1 Cell Collection**

The anesthesia was induced and the animals were prepared as described in section “Anesthesia and preoperative preparation”. After reaching the stage of surgical tolerance, male Lewis rats were euthanized by intracardiac injection of phenobarbital (Narcoren, Merial). The femora and tibiae were then removed directly from both hind legs under sterile conditions, the muscle, connective and cartilage tissue as well as the periosteum were carefully removed and placed in phosphate buffered saline (PBS). The proximal end of the femur and the distal end of the tibia were cut with scissors so that the larger knee joint surface could be used to insert the irrigation cannula. The bone marrow was cultured under aseptic conditions using 30ml cell culture medium (1:1 Ham's F12 (Carl Roth) & Dulbecco's Modified Eagle Medium (CELLPURE® Carl Roth) + 10% FCS + 1% P/S + 1% glutamine (Sigma Aldrich) ) rinsed out via a 21G irrigation cannula in the shaft of the bone. The cell particles of the bone marrow that were still connected were carefully aspirated several times with an injection needle and then slowly rinsed out again in order to separate the cells from one another. The bone marrow obtained in this way from a total of 4 long bones (2 femora and 2 tibiae) was divided into two 50 ml centrifuge tubes and each filled up to a volume of 50 ml with cell culture medium.

**1.2 Cell isolation**

The mononuclear cells were subsequently isolated using the density gradient principle (Ficoll protocol) (Boyum 1968). The cells isolated from the bone marrow were then further processed under sterile conditions. The two centrifuge tubes containing the cell suspensions were first centrifuged for 5 minutes at 368xg (Thermo Fisher Scientific, Heraeus Megafuge 16R, Osterode, Germany), then braked at speed 9, the supernatant was carefully aspirated and the cells were each washed with 25 ml of BMSC medium resuspended. The cell suspensions produced in this way were slowly applied dropwise using an electric pipette to 23 ml each of Ficoll (Ficoll-Paque™ plus, GE Healthcare, Sweden) in a 50 ml centrifuge tube. Both centrifuge tubes were then centrifuged at 3005 x g for 18 minutes. The braking function of the centrifuge remained switched off. Due to the different migration of the cells during the centrifugation, separate layers of the different cell groups were formed. After centrifugation, the upper layer of medium was carefully pipetted off without touching the underlying layer of mononuclear cells, which appeared cloudy. In addition to mononuclear cells, other slow-sedimenting particles such as thrombocytes are also found in this layer due to their density (Boyum 1968; Boyum 1976).

In the next step, the cloudy layer of mononuclear cells was carefully removed with a disposable pipette. The cells from both centrifuge tubes were then combined in a new 50 ml Falcon tube. This falcon tube was again filled up to 50 ml with fresh BMSC medium and centrifuged for 5 minutes at 368×g and then decelerated to brake level 9. The cells were then resuspended with 1000 μl of BMSC medium. To determine the cell concentration in the counting chamber (C-Chip Neubauer Improved disposable counting chamber, Roth, Karlsruhe), 1.0 μl of this cell suspension was mixed with 99 μl of a tryptan blue solution (C.I. 23850) (Carl Roth GmbH + Co. KG, Karlsruhe, Germany) diluted to 1:100 and incubated for three minutes at room temperature. The trypan blue solution was made up as a 0.5% solution containing 0.9% NaCl. The solution can be stored at room temperature for a few weeks. Apoptotic cells appear blue because the dye can enter through open channels of dead cells. The vital cells, on the other hand, do not accumulate the dye and appear light.

The counting chambers were counted under the microscope according to the formula adapted to the Neubauer counting chamber used (C-Chip Neubauer Improved Disposable counting chamber, Roth, Karlsruhe).

**2. FACS analysis of rBMMNC suspension for CXCR4, CXCR7 and CD45, CD90**

The isolated mononuclear cells were characterized using specific antibodies by fluorescence-activated cell sorting (FACS). The mononuclear stem cells isolated from the bone marrow of the donor animals were first stained with anti-erythrocyte medium (Life Technologies GmbH (Thermo Fisher Scientific), Darmstadt, Germany) and anti-granulocyte label (BD Bioscience, USA). This served to check the efficiency of the Ficoll protocol, i.e. the sorting out of erythrocytes and granulocytes. In addition, the surface proteins CD45-FITC (BioRad AbD Serotec GmbH, Puchheim, Germany) and C90-PE (BioRad AbD Serotec GmbH, Puchheim, Germany). The cells determined as CD45- & CD90+ were identified as BMSCs and thus represented the BMSC pool (Boxall and Jones 2012). In a further step, the mononuclear cells (lymphocytes, monocytes, etc.) were examined for the surface receptors CXCR4 (antibodies-online GmbH, Aachen, Germany), CXCR7 (antibodies-online GmbH, Aachen, Germany). CXCR4 and 7 are found on the surface of mononuclear cells (De Falco et al. 2004; Petit et al. 2007). Testing was performed using the FACSCantoTM II (BD Bioscience, USA).

Table 1 shows the preparation of the FACS buffer. Table 2 lists the steps of the staining protocol for FACS analysis.

**Table 1:** Composition FACS-Buffer

| **reagent** | **Manufacturer** | **composition** |
| --- | --- | --- |
| PBS (pH 7,4) |  |  |
| Bovine Serum Albumin | (Carl Roth GmbH + Co. KG, Karlsruhe, Deutschland) | 0.4% |
| sodium azide (NaN3) | (Carl Roth GmbH + Co. KG, Karlsruhe, Deutschland) | 0.02% |

To prepare the FACS buffer, bovine serum albumin (Carl Roth GmbH + Co. KG, Karlsruhe, Germany) at a concentration of 0.4% and sodium azide (NaN3) at a concentration of 0.02% were dissolved in PBS buffer and brought to a pH value of 7.4 set. BSA serves to reduce non-specific binding of the antibodies and sodium azide as a preservative. The solution was sterile filtered and stored in the refrigerator until use.

**Table 2:** FACS- staining protocol

| **step** | **method** |
| --- | --- |
| 1. | 1 × 106 cells in 50 μl FACS buffer into a centrifuge tube |
| 2. | Centrifuge the sample 1200 rpm, 5min, 4°C |
| 3. | remove supernatant |
| 4. | Incubate with the antibodies for 30 minutes at 4°C.   - CD45-FITC (Bio-Rad AbD Serotec GmbH, Puchheim, Germany): 1μl - CD90-PE (Bio-Rad AbD Serotec GmbH, Puchheim, Germany): 1:20 dilution, take 1µl - CXCR7-AK-APC (antibodies-online GmbH, Aachen, Germany): 2µl - CXCR4-AK-PE (antibodies-online GmbH, Aachen, Germany): 5 µl - Anti-Erythoid (Life Technologies GmbH (Thermo Fisher Scientific), Darmstadt, Germany): 1µl - PE maus anti-rat-granulocytes (BD Bioscience, USA): 1µl  \| **Tube** \| **Antibody** \| \| --- \| --- \| \| unmarked \| - \| \| 1 \| Anti-Ery + Anti-GZ \| \| 2 \| CD45-FITC + CD90-PE \| \| 3 \| CXCR7-APC + CXCR4-PE \|   Then fill samples with PBS to a total volume of 100 μl and mix with pulse vortex. |
| 5. | Centrifuge sample (350 x g, 5min, 4°C) |
| 6. | Remove supernatant and centrifuge resuspension in 1ml PBS |
| 7. | Centrifuge sample (350 x g, 5min, 4°C) |
| 8. | Remove supernatant and centrifuge resuspension in 1ml PBS |
| 9. | sample (350 x g, 5min, 4°C) |
| 10. | Resuspend cells in 100 μl PBS |

Vital and apoptotic cells (life-dead-staining) were marked shortly before the FACS analysis using the Sytox Blue Dead Cell Stain for flow cytometry (Invitrogen/Thermo Fisher Scientific, Germany) in a final dilution of 1:2000.

**3. Labeling with Qdot® 655 nanocrystals**

Fluorescence labeling of the living cells was carried out in vitro using Qdot® 655 nanocrystals (InvitrogenTM/ Thermo Fisher Scientific, Germany) in order to be able to subsequently quantify their adhesion or successful migration into the AVTECs by means of histological evaluation (Muller-Borer et al .2007). Component A contains the nanocrystals for cell marking and component B contains peptides that penetrate the cell membrane. The effectiveness of the in vitro cell labeling was confirmed by a FACS analysis.

Table 3 lists the individual steps of the labeling protocol.

**Table 3:** Protokoll Qtracker® Cell Labeling Kit

| **step** | **method** |
| --- | --- |
| 1. | To prepare 10 nmol/l labeling solution, 10 μl each of Qtracker® component A and component B were premixed in a 1.5 ml microcentrifuge tube. |
| 2. | Incubate for 5 min at room temperature |
| 3. | Add 200 μl BMSC medium and vortex mix for 30 sec |
| 4. | Add 1 x 107 cells |
| 5. | Incubate at 37°C for 45-60 min |
| 7. | Centrifuge sample, 5 min, 368 x g |
| 8. | Remove the supernatant and centrifuge the resuspension with 1000 μl BMSC medium |
| 9. | Centrifuge sample, 5 min, 368 x g |
| 10. | Remove supernatant and resuspend with 1000 μl BMSC medium |
| 11. | Centrifuge sample, 5 min, 368 x g |
| 12. | Remove supernatant and resuspend with 100 μl BMSC medium |
| 13. | visualize the cells under a fluorescence microscope or flow cytometry with the appropriate filters (excitation at 405-615 nm; emission at 655 nm) |

**4. Experimental animals**

syngeneic Lewis rats (Charles River, Sulzfeld, Germany) were used to carry out the in vivo experiments. The animals were taken over by the breeder when they were about 6 weeks old and weighed 220-240g. The animals were kept before and during the experiment under standardized conditions (constant room temperature of 22±2 °C with a humidity of 50±10%) and unlimited access to water and food (Ssniff Spezialdiaten GmbH, Soest, Germany). At least twice a week, the test animals were moved to a newly littered cage with fresh food and a water reservoir.

After taking over from the breeder, all animals were acclimatized for at least one week in groups of 5 animals. Only then did the surgical intervention begin. Chamber implantation was carried out on the animals with a weight of 290 to 340 g and an age of 7 to 9 weeks. In order to enable the animals to wake up relaxed in a quiet environment after the surgical intervention, one animal was placed in a cage with a floor area of ​​800 cm2 (cage type 3 H, Tecniplast Germany GmbH Hohenpeissenberg, Germany).

**5. Implant preparation**

**5.1. Teflon chambers**

Cylindrical Teflon chambers (Harhaus, Remscheid, Germany) manufactured by the Harhaus company were used for the implanted tissue construct. The chambers with an inner height of 7 mm and an inner diameter of 11 mm could be filled from above through a removable lid. The bottom, sidewalls and lid of the Teflon chambers were perforated with 2.0 mm diameter holes at regular close intervals. The fit of the lid was so high that simply tearing it out was enough for the defined closure. The titanium pins arranged in a square in the bottom of the chamber enabled standardized positioning of the AV loop that was inserted later and prevented its dislocation (Arkudas et al. 2009). A side wall of the chambers had a circular opening with a diameter of 4.0 mm, through which the vessels of the AVL could enter and exit the chamber. The Teflon chambers were adapted for the control group. For this, the side of the chamber opening and the opposite side of the chamber with a scalpel (size 22, Braun, Melsungen, Germany) notched. Through this slit, the separated saphenous artery and vein were able to enter and exit the chamber.

**5.2. Chamber contents**

The chamber was lined with 3 layers of the elastin-collagen matrix MatriDerm® (MedSkin Solutions, Dr. Suwelack AG, Billerbeck, Germany) and 1 ml collagen gel PureColTM EZ Gel for optimal support of the AV loop or the NON-AV loop (Sigma-Aldrich, USA). The PureColTM EZ Gel was stored in the refrigerator at a temperature of 4°C until use. 1 ml of the liquid was incubated at 37°C in an insulin syringe (1 ml) for 24 hours before the start of the operation to start gelation. Complete gelation was achieved after a period of 60-90 minutes, but increased stability of the gel was achieved through the extended incubation time.

First, two layers of MatriDerm® were placed in the chamber under sterile conditions, then the collagen gel was applied to the chamber under sterile conditions using an insulin syringe. 0.5 ml was introduced into the chamber floor below the loop, then the loop was inserted and then 0.5 ml was introduced above the loop. Before closing the chamber, another layer of MatriDerm® was applied.

**6. Anesthesia and preoperative preparation**

Before the beginning of the surgical interventions for the implantation of the AV loops, the non-AV loops and chambers, a detailed inspection and weight control of the animals was carried out. Anesthesia was induced in an induction box (UNO Roestvaststaal BV, Zevenaar, Netherlands) with an isoflurane concentration of 5.0% by volume (IsoFlo®, Ecuphar GmbH, Greifswald, Germany) and an O2 flow of 2 l/min. The animals were exposed to the gas conditions of the induction box for at least two minutes in order to achieve sufficiently deep anesthesia. After failure of the turning reflex, the rats were placed in a supine position on the 39°C warm and padded heating plate (UNO Roestvastaal BV, Zevenaar, Netherlands). Anesthesia was administered with spontaneous breathing using a semi-open anesthetic system with nasal mask and integrated exhaust hood (UNO Roestvastaal BV, Zevenaar, The Netherlands) with an isoflurane concentration of 1.5 to 2.5 vol% in pure oxygen (0.3 l/min ) maintained. The depth of anesthesia and reaching the surgical tolerance stages were checked on the basis of the clinical signs of respiration and heart rate and the failure of the interdigital reflex. In addition, about 30 minutes before the cut, the animals were given subcutaneous analgesia with 0.05 mg/kg body weight buprenorphine (Buprenovet®, Bayer Vital GmbH, Leverkusen, Germany). To prevent the conjunctiva from drying out during the operation, an eye ointment (Vit-A-Vision® eye ointment, OmniVision GmbH, Puchheim, Germany) was applied.

Before the surgical field was covered in a sterile manner, an indwelling venous catheter (Introcan Safety Winged 24G, Braun Melsungen AG, Melsungen, Germany) was placed in the lateral tail vein for intravenous application of 2 ml heparin solution (100 IU/ml) preoperatively and intraoperatively during the course of the operation.

The surgical field on the right and left hind leg was shaved and completely depilated using depilatory cream (Veet hair removal cream sensitive, Reckitt Benckiser (RB) Deutschland GmbH). The hind legs were fixed on the base in a stretched and slightly outwardly rotated position, disinfected with colorless skin disinfectant (Kodan Tincture forte, undyed, Schülke & Mayr GmbH, Norderstedt, Germany) and the operating area was covered with two sterile perforated adhesive towels (Foliodrape® Protect, Paul Hartmann AG, Heidenheim, Germany). The intraoperative monitoring of respiration and mucous membranes was carried out via a hole cut out in the drape at the level of the thorax and head.

**7. Surgical implantation of the chambers**

All operations with and without an AV loop were performed by the same surgeon (S.F.).

The surgical field was opened with a 3-4 cm incision of the skin with a scalpel on the inside of the left leg from the groin to the knee. On the muscle belly of the musculus gracilis, the vena and arteria saphena, as well as the nervus saphenus running parallel and laterally.

Using a surgical microscope (OPMI pico, Carl Zeiss Meditec AG, Jena, Germany), the subcutaneous tissue and fascia were first prepared in layers. This exposed the saphenous artery and vein. The dissection was made over a length of about 4 cm, from the outlet from the iliac vessels in the groin to the level of the knee joint. All of the vascular branches that could be visualized during the course of the operation were carefully coagulated using bipolar forceps and removed so that the vascular pedicles in the surgical area could be freely mobilized.

**7.1. Operation method of the experimental group with AV loop**

An arteriovenous vessel loop was generated in all animals of test group I and placed in the implantation chamber. First, an adventitectomy of the artery and vein at the later anastomosis sites was performed over a length of approx. 0.5 cm at the level of the distal thigh. The A. saphena was clamped proximally, shortly after its exit from the A. femoralis, with a microvascular clamp (B-1V, S&T AG, Neuhausen, Switzerland), placed at a 90° angle to the vessel axis. When selecting the height of the separation, care had to be taken to ensure sufficient distance to the vascular branches in order to prevent turbulent currents of the blood flow in the later anastomosis area. The vessel was then coagulated distally, slightly caudal to the knee, and severed at the site previously freed from adventita with a straight incision perpendicular to the vessel axis. It has already been shown in the literature that despite the interruption of the main vascular supply of the leg, no perfusion problems are to be expected distal to the vascular detachment (Huang et al. 1992; Schmidt et al. 2015). Using a vessel dilator, the vessel lumen was carefully dilated and rinsed with a heparin solution (100 IU/ml) diluted with NaCl (Heparin-Natrium Braun 50,000 IU/500 ml injection solution, B. Braun Melsungen AG, Melsungen, Germany) up to the level of the microvascular clamp and thus freed from microcoagulation. The saphenous vein in the groin area was clamped with a vascular clamp, caudally coagulated and severed. The stump of the vein was dilated in the same way and rinsed with heparinized solution.

Similar to the operation on the left side, the saphenous vein was dissected contralaterally. An approximately 15 mm long vein graft was removed from the right thigh and transferred to the surgical site. After the adventitectomy, the vein was coagulated proximally and distally with bipolar forceps. The vein graft was then removed with a smooth, straight incision perpendicular to the vessel axis. The ends of the vein piece were carefully widened with the vascular dilator and rinsed with heparinized solution (100 IU/ml) to remove blood clots. An anastomosis between the saphenous artery and the distal end of the vein graft and an anastomosis between the proximal end of the vein graft and the saphenous vein was then microsurgically created. The piece of vein was thus sewn in the reverse direction to avoid obstructing flow through the vein valves.

The anastomosis was sutured with eight to twelve single-button sutures, in which the vessel ends were connected end-to-end with an 11-0 nylon thread (Dafilon®, B. Braun Melsungen AG, Melsungen, Germany). In the literature, a proportion of less than 5% of the cardiac output is described for the perfusion of the AV loops. Therefore, no major blood pressure fluctuations or circulatory reactions of the animals were to be expected (Schmidt et al. 2015).

After completion of the arteriovenous and venovenous anastomosis, 1 ml heparin (100 IU/ml) was applied via the caudal vein. The vascular clamp of the vein was then released first. Only then was the hemostat removed from the arterial leg. The resulting arteriovenous shunt between the saphenous artery, vein graft and saphenous vein was clinically evaluated by inspection and smearing of the distal vein sections with regard to their patency and flow rate. Two drops of Paveron® (Paveron® N, 25 mg/ml solution for injection, Linden Arzneimittel-Vertrieb-GmbH, Heuchelheim, Germany) were applied near the vascular loop to dilate the vessels and resolve any existing vascular spasms. The patency of the loop and the tightness of the anastomoses were checked under the surgical microscope for at least 15 minutes. During the anastomosis control period, the isolation chamber was prepared for the implantation. For this purpose, the chamber was first cleaned with contact surface disinfection and then thoroughly rinsed with sterile NaCl solution.

The chamber was then half filled with 2 layers of MatriDerm® and then with 0.5 ml of PureColTM EZ Gel. The vessel loop was then carefully pulled through the largest circular opening in the chamber. Tension-free placement of the vessel loop around the four positioning pins and when entering and exiting the chamber. After checking the correct position, the chamber was filled with 0.5 ml PureColTM EZ Gel (Sigma-Aldrich, USA) and 1 layer of MatriDerm® and closed with the appropriate lid. The chamber was fixed to the fascia of the gracilis muscle with 2 sutures using a non-absorbable 6-0 polypropylene suture (Prolene®, Ethicon®, LLC., Puerto Rico, USA).

Finally, the wound was closed in two consecutive layers (subcutaneous & skin sutures) with absorbable 4-0 Vicryl suture (VicrylTM, Ethicon®, Johnson&Johnson International, Diegem, Belgium). The skin seam was cleaned with a moist compress, dried and additionally sealed with an aluminum spray (CP Pharma, Burgdorf, Germany).

**7.2. Operation method of control group without AV loop**

A Teflon chamber filled with MatriDerm® and collagen matrix was also implanted in all animals of the control group (group II), but without the construction of an arteriovenous vessel loop. Group II thus represents the non-axially vascularized control group.

A. and V. saphena were dissected immediately proximally after their exit from the femoral vessels to distally at the level of the knee joint. The vascular branches along this stretch were dissected free, coagulated and removed. As a result, the control group experienced an equally high burden of surgical trauma. For the control group, the design of the chambers was changed slightly. In order to be able to insert the vessels through the chamber, the chamber had to be slit at the large opening and on the opposite side using a scalpel. The chamber was also placed in the chamber with 2 layers of MatriDerm®, then with 0.5 ml of collagen gel (PureColTM EZ Gel, Sigma-Aldrich, USA) and then the saphenous artery and vein. The chamber was then completely filled with 0.5 ml collagen gel and a layer of MatriDerm®, closed with the lid and fixed to the underlying muscle with two single button sutures in opposite positions. The subcutaneous layer and skin were finally closed and trimmed as described above.

**7.3. Postoperative care**

Immediately after the operation, 1 ml of heparin (100 IU/ml) diluted in NaCl was injected intravenously via the indwelling vein catheter to prevent thrombosis. The catheter was then removed from the lateral tail vein. After the operation, the animals received a dose of 0.05 mg/kg bw buprenorphine (Buprenovet®, Bayer Vital GmbH, Leverkusen, Germany) injected subcutaneously every 12 hours for two days. The initial dose was applied subcutaneously immediately after skin closure. To prevent the animals from becoming chilled during recovery, they were placed in the right lateral position under a heat lamp and wrapped in a combination of cellulose and aluminum foil. Postoperative awakening took place under the constant supervision of the surgeon.

The condition of the animals in the postoperative course up to the day of removal of the construct was checked daily. In each case, a detailed wound check was carried out and the clinical condition of the animals was assessed on the basis of behavior, posture, coat and nutritional status. Due to the short postoperative examination period of 14 days, it was not necessary to remove the suture material.

**8. Stem cell injection**

In an AV loop model, vessel ingrowth begins between 10 and 14 days after implantation, depending on the matrix used (Polykandriotis et al. 2008). In addition, the initially high blood flow through the AV loop decreases to an average arterial flow about two weeks after implantation (Schmidt et al. 2015). In order to avoid a bias triggered by the initial non-physiologically high blood flow in the AV fistula, the marked stem cells were not injected in this study until 12 days after AVTEC implantation. 1 x 10^7^ freshly isolated and Qdot® 655 labeled rBMMNCs were injected slowly either intra-arterially (via the descending abdominal aorta exposed by a limited laparotomy) (groups Ia and IIa) or intravenously via the tail vein (groups Ib and IIb).

**8.1. Intra-arterial injection (group Ia, IIa):**

Inhalation anesthesia was initiated and maintained as previously described. The ventral abdomen of the animal was extended from the pubic symphysis to the level of the Processus xyphoideus electrically shaved and depilated with depilatory cream. After reaching the surgical tolerance stage, the abdomen was opened via a limited median laparotomy of about 2 cm in length. The intestinal loops were moved to the left and the abdominal aorta was exposed in the retroperitoneal space over a length of about 1 cm by blunt dissection in layers. 1 x 10^7^ stem cells isolated from a donor animal and labeled with Qdot® were dissolved in 1 ml BMSC cell culture medium and slowly injected into the abdominal aorta via an insulin syringe over a period of one minute. The cannula was rinsed with 1 ml NaCl. The cannula was removed and the puncture site compressed for two minutes. Hemostasis had to be secured before closing the abdominal incision in two layers with absorbable 4-0 Vicryl suture (VicrylTM, Ethicon®, Johnson&Johnson International, Diegem, Belgium). The skin seam was cleaned with a moist compress, dried and additionally sealed with an aluminum spray (CP Pharma, Burgdorf, Germany).

**8.2. Intravenous injection (Group Ib, IIb):**

Inhalation anesthesia was initiated and maintained as previously described. An indwelling venous catheter (Introcan Safety Winged 24G, Braun Melsungen AG, Melsungen, Germany) was placed in the lateral caudal vein. 1 x 107 stem cells isolated and labeled as described above and dissolved in 1 ml BMSC cell culture medium were injected slowly over this over a period of one minute. The cannula was rinsed with 1 ml NaCl to ensure complete delivery of the dose. The indwelling catheter was removed and the puncture site compressed for one minute.

**9. Sacrifice of animals and chamber explantation**

In order to ensure a stable distribution of the marked cells in all tissues, the reperfusion and subsequent explantation of the constructs took place 48 hours after injection of the marked stem cells (14 days after the operation) (Eggenhofer et al. 2014). To visualize and quantify the vascularity in the histological sections, the animals were exsanguinated and immediately reperfused with black calligraphy ink (Indian Ink).

**Table 4:** Composition of the ink solution for perfusion

| **reagent** | **Manufacturer** | **composition** |
| --- | --- | --- |
| 0,9% NaCl |  | 15 ml |
| Black-Indian-Ink | (Winsor & Newton, London HA3 5RH, England) | 15 ml |
| Gelatin | (Carl Roth GmbH + Co. KG, Karlsruhe, Deutschland) | 1,5g |
| Mannitol | (Carl Roth GmbH + Co. KG, Karlsruhe, Deutschland) | 1,2 g |

The induction and maintenance of inhalation anesthesia was carried out as previously described. First, the rat was shaved on the ventral abdomen and on the inner left thigh. For the exsanguination, the abdomen of the animals was opened along the entire length of the xyphoid process along the linea alba. The bundle of intestines was shifted out of the abdomen to the left and the abdominal aorta and the parallel caudal vena cava along the length of the outlet of the Aa. and Vv. renales up to the fork in the Aa. and Vv. Iliacae shown. Two ligatures (sewing thread, upper yarn mercerized, 100% cotton) were then presented. The first was looped around both vessels and placed slightly caudally, the second slightly more cranially and only around the abdominal aorta. The aorta was cannulated from cranial to caudal using a 24G venous catheter (Introcan Safety®-w, 24G×3⁄4”, 0.7×19 mm, yellow, B. Braun Melsungen AG, Melsungen, Germany) and this with the fixed aortic ligature. The caudal vein was perforated cranially to the ligature.

The animal was then perfused via the catheter with 100-150 ml of heparin-Ringer solution (100 IU/ml) heated to 39°C. Clear bleaching of the caudal areas of the animal confirmed that the flushing of the entire vascular system was carried out correctly. The rinsing process was terminated as soon as only clear liquid emerged from the V. cava caudalis. Then 30 ml of ink solution was infused (Kneser et al., 2006a). The infusion of the complete 30 ml Indian Ink solution was confirmed by a clear blackening of the animal, especially the caudal parts. The two vessels were then ligated caudally to the previously perforated site and the venous catheter was removed from the aorta. In this way, it was possible to prevent further leakage until the ink solution had completely hardened.

In order to ensure complete hardening of the ink-gelatin solution in the capillary vascular bed, the rat carcass was then stored at 4° C. for at least 12 hours or at -20° C. for at least 2 hours, and the chamber was then explanted. For this purpose, the left hind leg was opened over the operation scar with a scalpel and the chamber was dissected out. The organs liver, lungs, kidneys, spleen and bone marrow were also removed from the tibia of the left hind leg.

**10. Fixation of tissue samples**

The explanted chambers and the liver, lung, bone marrow and spleen tissues were fixed immediately after removal from the rat carcass. For this purpose, the tissue surrounding the chamber was severed sharply with a scalpel and the chamber was then placed in a 50 ml centrifuge tube containing approx. 20 ml 4% paraformaldehyde (PFA). The remaining tissues were also freed from fat and connective tissue and placed in a 50 ml Falcon tube with 30 ml 4% paraformaldehyde (PFA) so that they were adequately covered. The resulting reversible cross-linking of the proteins enables the tissue samples to be preserved. The fixation took place over 12 hours at a temperature of 4 °C.

**11. Preparation of histology**

**11.1 Dehydration and embedding of the tissue samples**

After fixing the constructs, the tissue samples were carefully dissected out of the Teflon chambers with a scalpel. The chamber lid was carefully loosened and lifted off. The four pins standing vertically in the chamber could be removed with a slight pull. The tissue samples were then dehydrated in an ascending alcohol series using the tissue infiltration device (Leica TP1020, Leica Biosystems Nussloch GmbH, Nussloch, Germany) (Table 5).

**Table 5:** Dehydration of the tissue samples

| **Station** | **Reagent** | **Temperature (°C)** | **Duration (min)** |
| --- | --- | --- | --- |
| 1 | 30% EtOH | 22 | 90 |
| 2 | 50% EtOH | 22 | 90 |
| 3 | 70% EtOH | 22 | 120 |
| 4 | 96% EtOH | 22 | 90 |
| 5 | 100% EtOH | 22 | 90 |
| 6 | 100% EtOH | 22 | 90 |
| 7 | 100% EtOH | 22 | 90 |
| 8 | 100% EtOH | 22 | 90 |
| 9 | xylene, 98% | 22 | 90 |
| 10 | xylene, 98% | 22 | 90 |
| 11 | Paraffin | 65 | 120 |
| 12 | Paraffin | 65 | 120 |

The now fully paraffinated cylindrical tissue samples of the chamber contents were divided into two equal halves along the diameter, perpendicular to the axis of the vascular pedicle. The constructs were divided into a proximal half with the vascular pedicle at the chamber entrance and a distal half with the vascular loop located in the chamber construct. The resulting halves were placed vertically in a metal mold with the cut surface facing down. This was followed by embedding with liquid paraffin heated to 60 °C. The tissues liver, spleen, right and left lung, right and left kidney and bone marrow were also embedded. The samples were clearly labeled and framed in embedding cassettes for later processing on the microtome. The paraffin blocks were completely cured and then devested from the molds on a deep-freeze surface (Leica Biosystems Nussloch GmbH, Nussloch, Germany).

**11.2 Sectioning technique**

For the preparation of the sections, the paraffin block had to be hardened on the cooling surface and cut until the entire extent of the tissue appeared on the sectioned surface. Sections with a thickness of 5 μm were made with a microtome (Leica RM2255, Leica Biosystems Nussloch GmbH, Nussloch, Germany). In order to maintain the cutting properties of the paraffin, the blocks had to be repeatedly stored on the deep-freeze surface between the individual cutting processes. The resulting sections were then stretched in a 40° C. water bath and mounted on a slide (Superfrost Ultra Plus, Gerhard Menzel GmbH, Braunschweig, Germany). In order to achieve good adhesion of the sections to the carrier, the sections were then laid on a warm surface at 40°C for a few minutes and then dried for 24 hours in a heating cabinet at an air temperature of 37°C.

**12. Histological and histomorphometric processing**

**12.1 Deparaffinization and rehydration**

After the sections on the slides had dried completely, they were deparaffinized with 98% xylene and then rehydrated as preparation for histological staining. The protocol is presented in Table 6.

**Table 6:** Deparaffinization und Rehydration protocol of the paraffin sections

| **Step** | **Reagents** | **Duration (min)** |
| --- | --- | --- |
| Deparaffinization | xylene, 98 % | 10 |
|  | xylene, 98 % | 10 |
| Rehydration | 100 % EtOH | 5 |
|  | 100 % EtOH | 5 |
|  | 95 % EtOH | 2 |
|  | 70 % EtOH | 2 |
|  | Aqua dest. | 1 (6x dips) |

**12.2 Histological staining**

**12.2.1 Hematoxylin-eosin stain**

For the initial morphological assessment of the patency of the AV loops, the paraffin sections of the chamber contents were stained with hematoxylin-eosin (HE) solution. The basic dye hematoxylin stains all basophilic cell structures (e.g. cell nuclei, mitochondria, collagen, elastin etc.) blue. The acidic eosin, on the other hand, stains all acidophilic components of the cell red. These include, among others, the cytoplasmic proteins, mitochondria, the smooth endoplasmic reticulum and collagen (Rentsch et al. 2014). The vascular lumina filled with India Ink appeared black in the histological examination and thus proved a patency of the arteriovenous vascular loop in group I or the detached vessels in group II.

**Table 7:** Hematoxylin-eosin staining protocol

| **Step** | **Reagents** | **Duration (min)** |
| --- | --- | --- |
| Hämatoxylin- staining | Hämatoxylin solution (Meyer’s) | 8 |
| Wash | Flowing tap water | 5 |
|  | 1 % HCl-Alcohol solution | 0.5 |
|  | Flowing tap water | 1 |
|  | 0.2 % ammonia solution | 0.5 |
|  | Flowing tap water | 5 |
| Eosin-staining | Eosin-solution | 5 |
|  | Flowing tap water | 1,5 (10-12x dips) |

**12.2.2 Hoechst 33342 - Propidium - Iodide (DNA staining)**

In fluorescence microscopy, the dye Hoechst 33342 is often used to counterstain the DNA and thus the cell nuclei of the sample. The fluorescent dye is excited at a wavelength of 350 nm, the emission of 461 nm is displayed with a blue fluorescence using a DAPI filter.

The final concentration of the staining solution should be 1μg/ml. The concentration of the prepared stock solution is 10mg/ml. The stock solution was stored at -20°C. A 1:5000 dilution was prepared from this by adding 2 μl of the Höchst stock solution to 9998 μl of distilled water. were resolved. A mark was drawn around the organ sections with a wax pencil so that the staining liquid could not run off the slide. Then 1 ml of the solution was applied to each section. The incubation took place for five minutes at room temperature in a light-protected box.

**12.2.3 Dehydration and coverslipping of the histological sections**

After staining, the sections were dehydrated in an ascending alcohol series. The protocol is presented in Table 8.

**Tabelle 8:** Protocol of ascending alcohol series

| **Step** | **Reagents** | **Duration (min)** |
| --- | --- | --- |
| Dehydration | Aqua dest. | 1 (6x dips) |
|  | 70 % EtOH | 1 |
|  | 95 % EtOH | 2 |
|  | 100 % EtOH | 5 |
|  | 100 % EtOH | 5 |
| Clearing | xylene, 98 % | 10 |
|  | xylene, 98 % | 10 |

After a final wash in xylene, the sections were then cleaned with a drop of the synthetic mounting medium (Roti®Histokitt II; Carl Roth GmbH + Co.KG, Karlsruhe, Germany) and coverslips (coverslips 24 × 50 mm, Carl Roth GmbH + Co. KG, Karlsruhe, Germany) are applied.

**13. Immunofluorescence staining against Hypoxia inducible Factor-1**

Deparaffinization and Rehydratation: **30 min**

| Deparaffinization | 98% xylene | 5 min |
| --- | --- | --- |
|  | 98% xylene | 5 min |
| Redydration | 100% ETOH | 5 min |
|  | 95% ETOH | 5 min |
|  | 70% ETOH | 5 min |
|  | 50% ETOH | 5 min |
|  | Aqua dest. | 2 min |

High Temperature Antigen Retrieval (**45 min)**

| 2ml Citrate-buffer (Antigen unmasking Solution /Vector) + 198ml Aqua dest. |
| --- |
| Slides in Citrate buffer into Wasserbad bei 98°C. Timer auf 10min stellen |
| Slides in Citrate buffer into 4°C and let it cool down to room temperature (circa 30 min) |
| Wash in PBS für 1 min |
| Wash again in PBS für 4 min |

Outline the tissue on the slides with hydrophobic pen (ImmEdge Pen, Vector Laboratories, H-4000).

Incubation with “Normal Blocking Serum”: **(35 min)**

| Incubation for 30 min in 3%BSA |
| --- |
| Wash in PBST for 1 min (0,1% Tween in 1%PBS) |
| Wash again in PBST for 4 min |

Incubation with primary-Antibody: (**overnight at 4°C)**

| Incubation of the non-control group with primary AB and the control group with PBS buffer. Incubation overnight at 4°C in the refrigerator (approx. 200μl per slide, dilution 1:200 with PBST) |
| --- |
| Wash in PBST for 1 min |
| Wash again in PBST for 4 min |

Dehydration & coverslips: (**35 min)**

| Dehydratation | 70% EtOH | | 5 min |
| --- | --- | --- | --- |
|  | 95% ETOH | | 5 min |
|  | 100% ETOH | | 5 min |
|  | 100% ETOH | | 5 min |
|  | 98% xylene | | 5 min |
|  | 98% xylene | | 5 min |
| Covering with Histokitt & coverslips | |  |  |

The analysis of the immunofluorescence staining took place using a Zeiss Vision microscope with the Rhodamine filter with an excitation of 540-552 nm and an emission of 575-640 nm.

**14. Fluorescence Microscopy**

Using fluorescence microscopy, the lung, liver, spleen and bone marrow tissues were examined for the stem cells that had previously been labeled and injected using Qdot® 655 nanocrystals (Invitrogen/ Thermo Fisher Scientific, Germany). The fluorescence microscope Zeiss Axio Imager.M2 (PI 10x/23) from Carl Zeiss was used.

According to the manufacturer's information, the Qdot® 655 can be excited at a wavelength in the range of 405-615 nm and have a relatively constant emission of 655 nm. Counterstaining of the DNA in the cell nuclei with Hoechst 33342 was detected at 350 nm using a DAPI filter. The quantum dots appear brightly fluorescent and aggregated in clumps in the individual cells. This could be demonstrated in a cell culture.

**15. Histological and histomorphometric evaluation**

The distribution of the Qdot-marked cells as a whole and their relationship to the vessel axis was histologically evaluated. In a first step, the total area of ​​the specimen was divided into 8 fields. These in turn were divided into a peripheral and a central part. The central part represents the area that appears in the field of view of the eyepiece at a magnification of 100x if the vessel is defined as the center. The number of Qdots was determined centrally, peripherally and in total at five proximal cuts and five distal cuts. Consequently, a total of 10 sections were evaluated per chamber and animal.

The evaluation was carried out directly on the microscope (Axioimager 2, Carl Zeiss), since the light signals from the Qdots were weakened during automatic recording by the microscope system and could not be displayed with all clarity by the program.

Liver and spleen tissues were evaluated per 10 fields of view at 200x magnification and the Qdots per field of view were counted. The counting took place in 8 visual fields, the mean value was formed.

Since the tissue sections of the spleen and liver were evaluated at a magnification of 200x and the chamber at a magnification of 100x, the density of the distribution of the Qdots (Qdots/mm2) must be calculated in order to be able to compare them with one another. To do this, use the calculation of the mapped area of ​​a circle using the field number. The area of ​​the circle is defined as A = π r². The lens used (10x/23) has a field number of 23. The field number defines the size of the field of view that is visible to the observer. Consequently, the larger the value, the more specimen surface the observer can see. With a field number of 23 and a 100x magnification, the diameter of the depicted circular area of ​​the specimen corresponds to d=2.3mm and thus the radius (r = 0.5 × d) r=1.15mm. Analogously to the above formula, this then corresponds to an imaged area of ​​4.154 mm2. With a field number of 23 and a 200x magnification, the diameter corresponds to d= 1.15 mm and thus the radius (r=0.5×d) r= 0.575 mm. Analogous to the above formula, this then corresponds to an imaged area of ​​1.0386 mm2.

**16. Statistical evaluation**

The quantitative analysis of the resulting tissue and vascularization, the HIF-1 expression and the accumulation of the fluorescently labeled stem cells in the chambers was presented in the form of the arithmetic mean and the standard deviation. The values ​​of the two groups (intra-arterial vs. intravenous injection) are compared using the unpaired t-test. For all statistical tests used, p-values ​​<0.05 are reported as statistically significant. If there is a p-value that is greater than this value, the significance level is missed. The p-values ​​obtained are interpreted purely descriptively and have no confirmatory value.

The software GraphPad Prism 8 for Mac was used. The statistical evaluation was carried out by Ms. Flechtenmacher herself. The results are presented as a bar chart showing the standard deviation and the p-value.
